# Variant-proof high affinity ACE2 antagonist limits SARS-CoV-2 replication in upper and lower airways

**DOI:** 10.1038/s41467-024-51046-w

**Published:** 2024-08-12

**Authors:** Matthew Gagne, Barbara J. Flynn, Christopher Cole Honeycutt, Dillon R. Flebbe, Shayne F. Andrew, Samantha J. Provost, Lauren McCormick, Alex Van Ry, Elizabeth McCarthy, John-Paul M. Todd, Saran Bao, I-Ting Teng, Shir Marciano, Yinon Rudich, Chunlin Li, Shilpi Jain, Bushra Wali, Laurent Pessaint, Alan Dodson, Anthony Cook, Mark G. Lewis, Hanne Andersen, Jiří Zahradník, Mehul S. Suthar, Martha C. Nason, Kathryn E. Foulds, Peter D. Kwong, Mario Roederer, Gideon Schreiber, Robert A. Seder, Daniel C. Douek

**Affiliations:** 1 Vaccine Research Center, National Institute of Allergy and Infectious Diseases, National Institutes of Health, Bethesda, MD USA; 2 Bioqual Inc., Rockville, MD USA; 3 Department of Biomolecular Sciences, Weizmann Institute of Science, Rehovot, Israel; 4 Department of Earth and Planetary Sciences, Weizmann Institute of Science, Rehovot, Israel; 5 Center for Childhood Infections and Vaccines, Children’s Healthcare of Atlanta, Division of Infectious Diseases, Department of Pediatrics, Emory University School of Medicine, Atlanta, GA USA; 6 Emory Vaccine Center, Emory University, Atlanta, GA USA; 7 Emory National Primate Research Center, Atlanta, GA USA; 8 Department of Microbiology and Immunology, Emory University, Atlanta, GA USA; 9 Biostatistics Research Branch, Division of Clinical Research, National Institute of Allergy and Infectious Diseases, National Institutes of Health, Bethesda, MD USA; 10 Present Address: Fred Hutch Cancer Center, Seattle, WA USA

**Keywords:** Viral infection, SARS-CoV-2, Applied immunology, Mucosal immunology, Drug discovery

## Abstract

SARS-CoV-2 has the capacity to evolve mutations that escape vaccine- and infection-acquired immunity and antiviral drugs. A variant-agnostic therapeutic agent that protects against severe disease without putting selective pressure on the virus would thus be a valuable biomedical tool that would maintain its efficacy despite the ongoing emergence of new variants. Here, we challenge male rhesus macaques with SARS-CoV-2 Delta—the most pathogenic variant in a highly susceptible animal model. At the time of challenge, we also treat the macaques with aerosolized RBD-62, a protein developed through multiple rounds of in vitro evolution of SARS-CoV-2 RBD to acquire 1000-fold enhanced ACE2 binding affinity. RBD-62 treatment equivalently suppresses virus replication in both upper and lower airways, a phenomenon not previously observed with clinically approved vaccines. Importantly, RBD-62 does not block the development of virus-specific T- and B-cell responses and does not elicit anti-drug immunity. These data provide proof-of-concept that RBD-62 can prevent severe disease from a highly virulent variant.

## Abstract (web summary)

SARS-CoV-2 evolution poses a risk to vaccine and antiviral drug efficacy. Here, Gagne et al. report the development of a variant-agnostic protein, RBD-62, with enhanced ACE2 binding obtained through in vitro evolution and show that RBD-62 inhalation protects nonhuman primates against SARS-CoV-2 Delta challenge.

## Introduction

SARS-CoV-2 variants of concern (VOC) including B.1.351 (Beta), B.1.617.2 (Delta) and the currently circulating sublineages of B.1.1.529 (Omicron) have acquired mutations that enable substantial escape from neutralizing antibodies in convalescent or vaccine sera^1–7^. Efficacy against severe disease after two doses of mRNA COVID-19 vaccines has declined from ~100% in clinical trials conducted at a time when ancestral strains were predominantly in circulation to 60–80% during the Omicron BA.1 wave^8–12^, a result of both waning antibody titers and virus-acquired mutations. Boosting can restore protective efficacy but the benefit of boosting beyond a third dose is unclear^13–15^ and accumulating evidence suggests that antigenic imprinting may offset the benefit of variant-matched boosts^16–21^.

Anti-viral therapeutic agents can reduce the effects of severe COVID-19 in individuals with or without prior immunity. Two drugs granted emergency use authorization by the FDA include Merck’s molnupiravir (Lagevrio) and Pfizer’s nirmatrelvir/ritonavir (Paxlovid). Interim data indicated that molnupiravir, a cytidine analog prodrug, reduced hospitalizations from COVID-19 by about 50% but further analysis has suggested a lower efficacy^22^. Nirmatrelvir/ritonavir has demonstrated substantial clinical efficacy, with an 89% decline in severe disease^23^. However, nirmatrelvir/ritonavir, which functions by inhibiting the main protease, is not routinely prescribed for the treatment of COVID-19^24,25^. Furthermore, the emergence of drug-resistant mutations in the virus remains a possibility; while some have already been detected in people, no widely circulating variants currently demonstrate this capacity^26–29^. Nonetheless, concerns regarding virus escape and the possibility for reduced anti-viral potency against emerging SARS-CoV-2 strains continue to elicit significant interest in the development of more effective protease inhibitors^30,31^.

Consequently, there remains an urgent need for the development of additional therapeutic agents that reduce severe disease, particularly those that act in a variant-agnostic manner; that is, without directly targeting the virus. Host-targeted approaches could maintain their efficacy as new variants emerge even if they were so divergent from the ancestral strains such that anti-viral drugs or prior immunity were rendered ineffective. We have previously described the development of an in vitro mutated SARS-CoV-2 receptor-binding domain (RBD) that displays greatly enhanced binding to the virus target receptor, angiotensin-converting enzyme 2 (ACE2), without inhibiting its natural enzymatic activity (Supplementary Table 1). Wildtype RBD was exposed to successive iterations of error-prone PCR which allowed for the selection of proteins capable of binding increasingly lower concentrations of ACE2 followed by pre-equilibrium selection to obtain faster association. The final product, termed RBD-62, has a binding affinity for ACE2 of 16 pM, an increase of 1000-fold compared to the wildtype Wuhan-Hu-1 (WT) RBD, which has a binding affinity of 1700 pM^32^. In vitro models demonstrated its capacity to block infection of cell lines with a half-maximal inhibitory concentration (IC_50_) of 18 pM against the Beta variant. RBD-62 treatment of Syrian hamsters through inhalation at the time of infection with the ancestral strain USA-WA1/2020 (WA1) also resulted in protection against weight loss. Protection against severe disease in a model system more closely approximating humans or in the context of infection from a VOC that has been shown to result in substantial morbidity and mortality has previously not been established.

Here, we infected rhesus macaques with Delta which is the most pathogenic variant tested to date in these animals. Macaques were treated immediately prior to challenge and every 24 h for the next 5 days with RBD-62 administered to the airways via aerosolization. Protection was measured via titers of culturable virus and subgenomic virus RNA (sgRNA). We also analyzed mucosal and serum immune responses to Delta-specific antigens as well as to RBD-62 itself to assess any anti-drug antibody (ADA) responses.

## Results

### RBD-62 inhibits binding between the SARS-CoV-2 spike (S) and ACE2 in a variant-agnostic manner

Efficacies for COVID-19 therapies and vaccines have declined in the context of emerging variants, which has limited the long-term applicability of results established in initial clinical and pre-clinical research. To determine if data gathered on RBD-62 from a challenge model with an ancestral variant could be extrapolated to current and future emerging strains, we first used an in vitro assay to examine the ability of RBD-62 to block binding between ACE2 and S from a panel of different variants.

**Fig. 1:**
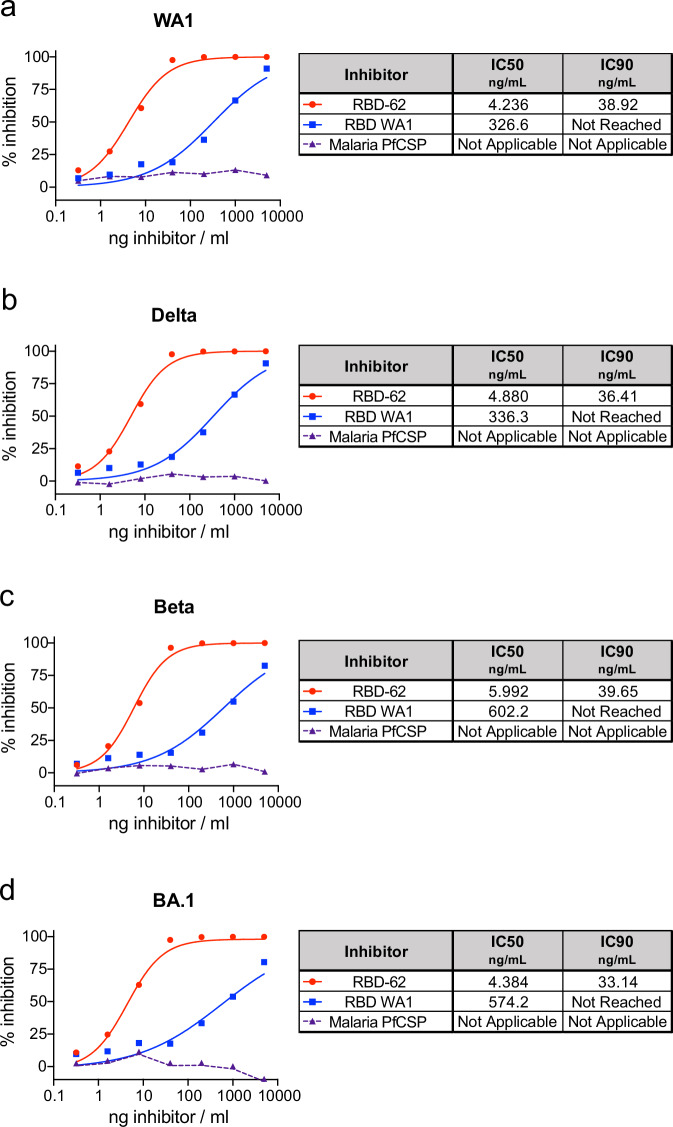
Inhibition of variant S–ACE2 binding. SARS-CoV-2 S from WA1 (**a**), Delta (**b**), Beta (**c**) and BA.1 (**d**) were mixed with soluble ACE2 in combination with indicated concentrations of RBD-62, RBD from WA1 or an irrelevant malaria protein (PfCSP) to determine percentage binding inhibition relative to maximum binding without inclusion of inhibitor. Icons represent the average inhibition of duplicate technical replicates at each indicated dilution. IC_50_ and IC_90_ values (ng/mL) are indicated to the right of each graph and, along with the curves presented within the graphs, were calculated using the nonlinear regression analysis tool in Prism. The dotted lines indicate background inhibition observed for PfCSP. Source data are provided as a Source Data file.

In contrast to unmutated WA1 RBD, which inhibited binding of both WA1 S and Delta S with an IC_50_ of ~330 ng/mL, RBD-62 was nearly 100 times more potent with IC_50_ values of ~4.5 ng/mL (Fig. 1a, b). Further, RBD-62 blocked binding between ACE2 and BA.1 S at almost the same concentration, and the IC_50_ for Beta S was only modestly higher at 6 ng/mL (Fig. 1c, d). Strikingly, IC_90_ values for RBD-62 against all variants were <40 ng/mL, whereas we were unable to achieve 90% binding inhibition for any variant using WA1 RBD as the inhibitor at any of our tested concentrations.

We further characterized the ability of RBD-62 to directly inhibit infection of VeroE6-TMPRSS2 cells with authentic WA1 and BA.1 viruses in addition to the more recently circulating Omicron sublineages BA.5, XBB.1.5 and JN.1. Similar to our findings on ACE2 binding, RBD-62 blocked in vitro infection at 100-fold lower concentrations compared to ancestral WA1 RBD, although the amount of protein necessary to achieve 50% inhibition was greater for infection than for ACE2 binding (Supplementary Fig. 1). As the potency of RBD-62 to block the ability of the virus to engage its receptor and infect target cells was strongly preserved across variants despite large differences in S sequence and binding affinity, we proceeded with in vivo evaluation of this product in a challenge model with Delta which, in our experience, is the most virulent variant and replicates at a high titer in the upper and lower airways of rhesus macaques.

### RBD-62 protects rhesus macaques from Delta replication in the upper and lower airways

We delivered 2.5 mg RBD-62 to eight rhesus macaques as an aerosol using the PARI eFlow® nebulizer as described previously^33^ to target the drug to both the upper and lower airways. In addition, a further eight macaques received aerosolized PBS control. Both groups were challenged 1 h later with Delta at a dose of 2 × 10^5^ median tissue culture infectious dose (TCID_50_). Primates continued to receive the same dose of aerosolized RBD-62 or PBS once per day for the next 5 days at which point treatment was stopped so that we could track the kinetics of virus rebound. Nasal swabs (NS) and bronchoalveolar lavage (BAL) were collected on days 2, 4, 7, 9 and 14, and RNA was isolated for detection of virus replication by PCR for sgRNA encoding for the virus nucleocapsid (N) transcript (Supplementary Fig. 2).

**Fig. 2:**
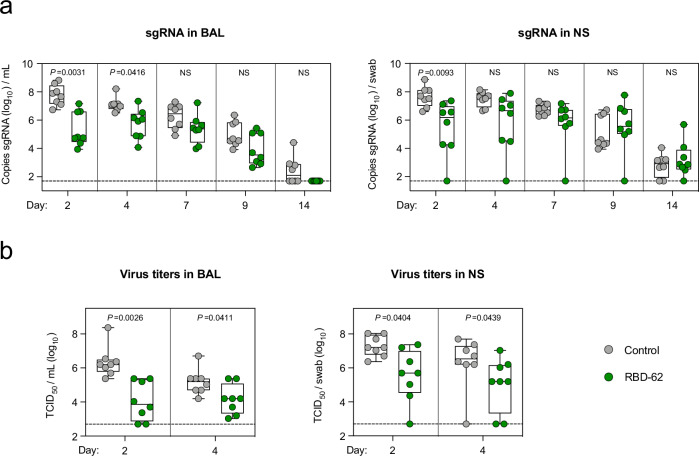
Delta replication in upper and lower airways. NHP (*n* = 8/group) were challenged with 2 × 10^5^ TCID_50_ Delta and simultaneously treated with RBD-62 (green circles) or PBS (gray circles). **a** Subgenomic RNA encoding for N transcript was measured in the upper and lower airways at days 2, 4, 7, 9 and 14 post challenge. **b** Culturable virus was measured in the upper and lower airways at days 2 and 4 post challenge. Dotted lines indicate the assay limit of detection (LOD). Circles, boxes and horizontal lines represent individual animals, interquartile range and median, respectively, while minima and maxima are denoted at whisker termini. Statistical analyses were shown for comparison of groups at each timepoint and were performed using the Wilcoxon rank-sum test (two-sided) after Holm’s adjustment across timepoints. NS denotes that the indicated comparison was not significant, with *P* > 0.05. See also Supplementary Tables 2 and 3 for complete statistical analyses. Source data are provided as a Source Data file.

We observed a significant decrease in virus sgRNA copies in the lungs on day 2 with a geometric mean of 2.3 × 10^5^ copies/mL BAL in the RBD-62 treatment group and 5.8 × 10^7^ copies in the PBS control group (*P* = 0.0031). Likewise, RNA copies in the nose on day 2 were similar to the BAL, with geomeans of 2.8 × 10^5^ copies/swab in the treatment group and 4.5 × 10^7^ in the control group (*P* = 0.0093) (Fig. 2a and Supplementary Table 2). Interestingly, the protective effect was no longer significant in either the nose or the lungs by day 7, which was the first collection timepoint after the cessation of RBD-62 treatment (*P* > 0.05). Nonetheless, peak sgRNA copy numbers in the RBD-62 cohort were lower than in the control primates. For instance, while 4/8 control NHP had peak copy numbers >10^8^ in either the lungs or the nose, virus titers never reached that level in any of the RBD-62-treated animals. Further, virus was cleared from the lower airway of all animals in the RBD-62 group by day 14 post challenge whereas half of the control group still had detectable sgRNA at that timepoint.

We also measured culturable virus via TCID_50_ which could indicate the potential for transmissibility (Fig. 2b and Supplementary Table 3). On day 2, the RBD-62 group had significantly less culturable virus than control animals, with geometric mean TCID_50_ values in the lungs of 1.1 × 10^4^ and 2.2 × 10^6^, respectively (*P* = 0.0026). Culturable virus was also reduced in the upper airway, with TCID_50_ of 3.6 × 10^5^ for the RBD-62 group and 1.9 × 10^7^ for the control group (*P* = 0.0404). As we kept all animals alive for 2 weeks to enable longitudinal analysis of virus clearance, we were not able to measure pathology or clearly identify virus antigens in tissues, which would have been most evident shortly after the challenge.

### Treatment with RBD-62 does not inhibit the induction of anti-SARS-CoV-2 humoral immunity

**Fig. 3:**
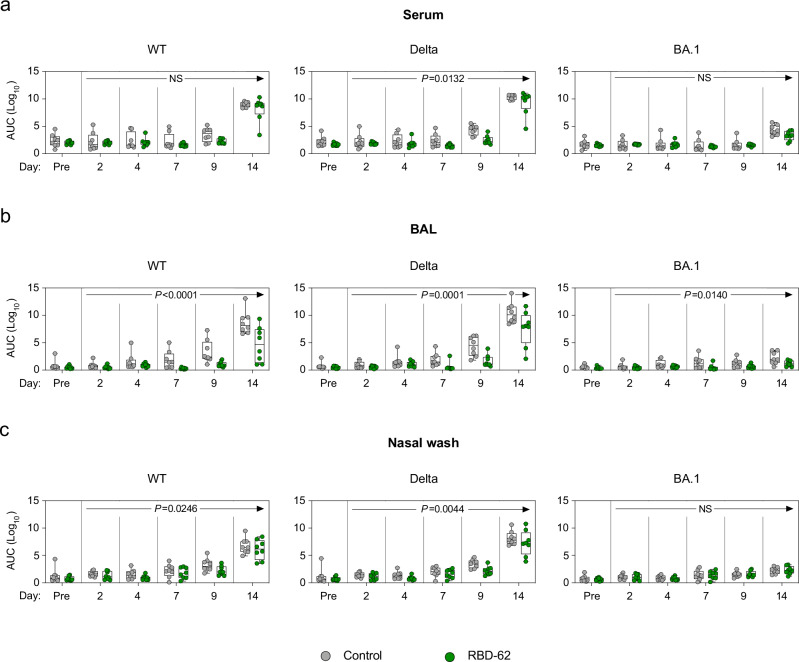
Anti-SARS-CoV-2 IgG binding titer kinetics. NHP (*n* = 8/group) were challenged with 2 × 10^5^ TCID_50_ Delta and simultaneously treated with RBD-62 (green circles) or PBS (gray circles). IgG binding titers were measured to wildtype, Delta and BA.1 RBD in **a** serum, **b** BAL and **c** nasal wash 1 month prior to challenge (pre-challenge) and on days 2, 4, 7, 9 and 14 post challenge. Serum was initially diluted 1:100 and then serially diluted 1:4. BAL and NW samples were initially diluted 1:5 and then serially diluted 1:5. Circles, boxes and horizontal lines represent individual animals, interquartile range and median, respectively, while minima and maxima are denoted at whisker termini. *P* values annotated on plots can be used to assess the statistical significance of a drug-specific treatment effect (difference between RBD-62 and control groups), based on two-sided generalized estimating equation (GEE) modeling, which included titers across all post challenge timepoints. No adjustments were made for multiple comparisons. NS denotes that the indicated comparison was not significant, with *P* > 0.05. See also Supplementary Table 4 for complete statistical analyses. Source data are provided as a Source Data file.

It is conceivable that the use of an ACE2-binding inhibitor during acute SARS-CoV-2 infection could prevent the formation of a primary or secondary immune response to the virus which would have been beneficial in the context of a future exposure. To test this hypothesis, we measured serum and mucosal IgG binding titers to a panel of variant RBDs, including WT, Delta and Omicron BA.1. At day 14 following the challenge, titers to the Delta challenge stock were greater than either of the other strains for both the treated and untreated animals, indicative of a primary response. Geometric mean titers (GMT) to Delta rose from a baseline of 5 × 10^1^–2 × 10^9^ area under the curve (AUC) in the serum of the RBD-62 group by day 14 (Fig. 3a). While we observed a similar increase in GMT of control NHP by day 14, from 2 × 10^2^ to 2 × 10^10^ AUC, the kinetics were faster with evidence of a primary response as early as day 9. We next confirmed that there was a differential treatment effect across the entire 14-day time course, which would indicate a blunted primary response due to a reduction in virus antigen in the RBD-62 group compared to the untreated group (Supplementary Table 4). Indeed, there was a significant treatment effect, with *P* = 0.0132. Likewise, mucosal binding titers to Delta RBD were higher on day 14 in the control primates, with GMT of 3 × 10^10^ in the lungs and 2 × 10^8^ in the nose as compared to 4 × 10^7^ and 2 × 10^7^ in the treated NHP respectively (*P* = 0.0001 in lungs and 0.0044 in nose) (Fig. 3b, c). Despite the attenuated response in the treated group, which reflected greater virus control by these animals, RBD-62 administration did not preclude seroconversion.

Although treated animals developed antibodies to variant RBDs, it is possible that this response largely reflected or was enhanced by anti-drug immunity as RBD-62 is itself derived from WT RBD. To confirm that the immune response was not limited only to RBD, we quantified binding to various proteins and domains including RBD, whole S and nucleocapsid (N). Further, as we focused our analysis on WT protein, we reported responses using the WHO-determined standard antibody units for WT virus. We detected evidence of a primary response to RBD and S in the serum and BAL of control animals by day 9, while binding titers in the treated animals were not noticeably greater than background until day 14 (Supplementary Fig. 3a, b). Similarly, animals in the RBD-62-treated group mounted primary responses to WT nucleocapsid but with delayed kinetics and decreased magnitude as compared to the control cohort. On day 14, anti-nucleocapsid GMT reached 11.4 antibody units per mL (BAU/mL) in the serum and 0.1 in the BAL of controls compared to 3.5 and 0.01 in the RBD-62 group. Titers in the nasal wash (NW) were markedly lower for both groups than in the BAL or serum (Supplementary Fig. 3c).

To further explore the effect of RBD-62 on the development of mucosal responses, we measured IgA binding titers to the aforementioned proteins (Supplementary Fig. 4). In agreement with our findings on IgG, the kinetics of the IgA response were faster in controls than in treated animals, with evidence of increased titers to RBD and S in the BAL of the controls by day 9 compared to day 14 in the RBD-62 group. However, we were nonetheless able to detect the binding of IgA to both RBD and S in the BAL and NW of the RBD-62 group. Mucosal IgA responses to N were not clearly above background for either group of animals. Together with the data on mucosal IgG responses, this would suggest that a future mucosal vaccine boost would likely not be affected by prior RBD-62 treatment.

We have previously used the ACE2–RBD binding inhibition assay as a surrogate for neutralization in the mucosa^16,34,35^. Here, we were able to detect inhibition of WT and Delta variants in the BAL of both control and treated groups (Supplementary Fig. 5a). We calculated that the median inhibitory capacity of BAL antibodies from the RBD-62 group was 6% against WT RBD and 8% against Delta RBD. Omicron BA.1 RBD–ACE2 binding inhibition was not detected, likely due to the divergence between the challenge stock and BA.1. NW inhibitory antibodies were not detectable in either group of animals (Supplementary Fig. 5b).

### Treatment with RBD-62 does not inhibit the induction of anti-SARS-CoV-2 T cell responses

**Fig. 4:**
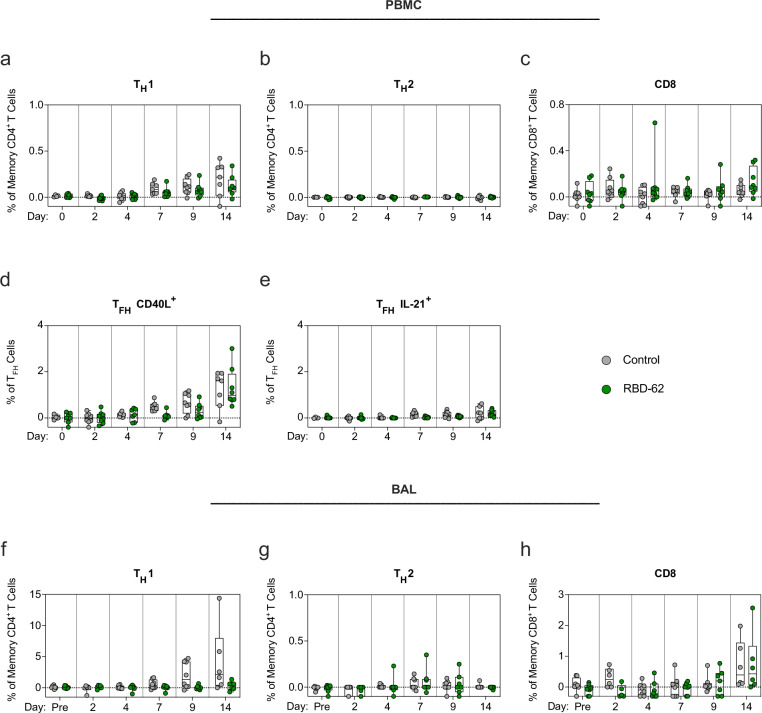
Kinetics of primary T cell responses following Delta challenge. NHP (*n* = 8/group) were challenged with 2 × 10^5^ TCID_50_ Delta and simultaneously treated with RBD-62 (green circles) or PBS (gray circles). **a**–**e** Peripheral blood mononuclear cells (PBMC) or **f**–**h** lymphocytes from BAL were collected prior to challenge (immediately preceding challenge for PBMC and 1 month pre-challenge for BAL) and on days 2, 4, 7, 9 and 14 post challenge. Cells were stimulated with WA1 S1 and S2 peptide pools and responses were measured by intracellular cytokine staining (ICS). **a**, **f** Percentage of memory CD4^+^ T cells expressing T_H_1 markers (IL-2, TNF or IFNγ). **b**, **g** Percentage of memory CD4^+^ T cells expressing T_H_2 markers (IL-4 or IL-13). **c**, **h** Percentage of memory CD8^+^ T cells expressing IL-2, TNF or IFNγ. **d**, **e** Percentage of T_FH_ cells expressing CD40L or IL-21, respectively. Dotted lines set at 0%. Reported percentages may be negative due to background subtraction and may extend beyond the lower range of the *y*-axis. Circles, boxes and horizontal lines represent individual animals, interquartile range and median, respectively, while minima and maxima are denoted at whisker termini. Due to pre-specified minimum cell numbers per sample required for analysis, some timepoints include data from <8 NHP/group. Data are provided as a Source Data file.

T cell epitopes present within SARS-CoV-2 are highly conserved^16,36^, suggesting that while newly emerging variants may continue to escape humoral immune responses, protection arising from T cell immunity may still be preserved. Thus, we next measured T cell responses to WT S peptides to determine if the administration of RBD-62 would interfere with their induction (Supplementary Fig. 6). Again, the kinetics of this response were faster in the control animals, with measurable increases in T_H_1 and CD40L^+^ T_FH_ responses in the periphery by day 7 compared to day 9 in the treated group (Fig. 4a–d). S-specific T_H_1 responses reached a median frequency of 0.2% in the controls and 0.1% in the treated NHP by day 14 although the difference was greater in the BAL (Fig. 4f). We did not detect T_H_2 responses in either the circulation or BAL (Fig. 4b, g). While we were able to detect some S-specific CD8^+^ T cells in the circulation of both groups, responses were much higher in the lungs—the primary site of virus replication—with median frequencies of 0.4% in both groups (Fig. 4c, h). We were also able to detect T cell responses to N in the circulation of the RBD-62 group (Supplementary Fig. 7).

### RBD-62 administration does not impair B-cell memory to SARS-CoV-2 and does not elicit anti-drug immunity

**Fig. 5:**
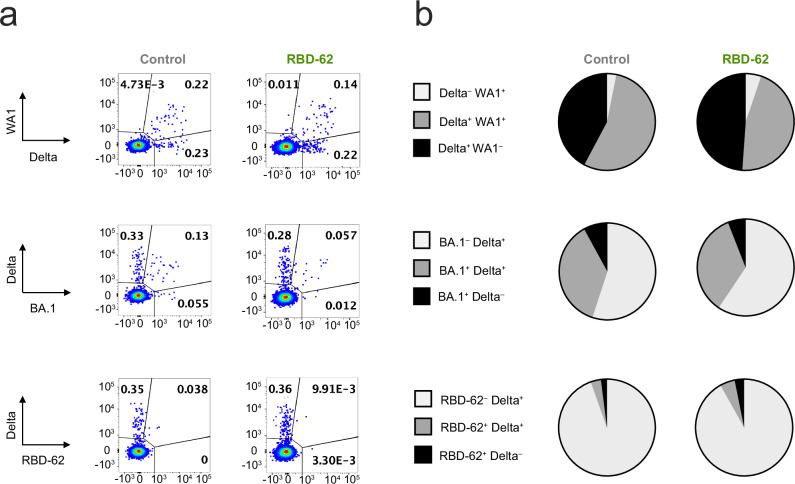
Memory B-cell responses following Delta challenge and RBD-62 treatment. NHP were challenged with 2 × 10^5^ TCID_50_ Delta and simultaneously treated with RBD-62 or PBS. Memory B-cell specificity was determined at day 14 post challenge via binding to fluorochrome-labeled variant probe pairs as indicated in figure legends. Probe pairs include WA1 and Delta S-2P, Delta and BA.1 S-2P, and Delta S-2P and RBD-62. **a** Representative flow cytometry graphs for one animal in the control group (left) or treated group (right). Event frequencies denote the proportion of probe-binding cells within the total class-switched memory B-cell population. Cross-reactive memory B cells are represented by events in the top right quadrant whereas single-positive memory B cells reside in the top left or bottom right quadrants. **b** Pie charts indicating the geometric mean frequency of the entire S-specific memory B-cell compartment capable of binding to both members of a variant probe pair (dark gray) or a single variant within the pair (light gray or black) at day 14 post challenge. The control group is displayed on the left and the treated group is displayed on the right. *n* = 8 for treated group and *n* = 4 for control group. Source data are provided as a Source Data file.

Memory B cells are essential for mounting secondary responses upon boosting or reinfection and, together with long-lived plasma cells, form the basis of long-term humoral immunity^37–40^. Due to imprinting, the initial exposure to virus establishes B-cell antigen specificity and determines the capacity of the immune system to recognize novel variants^16–21^. We therefore collected memory B cells from the peripheral circulation on day 14 following challenge and measured binding to pairs of fluorescently labeled variant S-2P probes (Supplementary Fig. 8). Control and RBD-62-treated animals displayed almost identical patterns (Fig. 5a, b); out of all Delta and/or WA1-binding memory B cells in the controls, a geometric mean frequency of 42% bound to Delta alone compared to 49% in the treated animals. In the controls, 55% of the population was cross-reactive and capable of binding both S compared to 46% in the RBD-62 group. When examining the pool of memory B cells capable of binding to Delta and/or BA.1, only 37% and 35% were dual-specific in the controls and the RBD-62 group, respectively. This smaller fraction of BA.1/Delta-specific cross-reactive cells as compared to the WA1/Delta-specific cross-reactive pool is likely due to the reduced number of shared epitopes between BA.1 and Delta. We also measured the frequency of S-specificity among all memory B cells (Supplementary Fig. 9). As most recently circulating SARS-CoV-2 strains are within the Omicron lineage, any limitation that RBD-62 treatment would place on the development of BA.1-binding B-cell responses would be especially concerning. However, the frequencies of BA.1/Delta-specific cross-reactive memory B cells were similar between the two cohorts with geometric mean frequencies of 0.14% in the controls and 0.13% in the treatment group (Supplementary Fig. 9b).

We next expanded our analysis of memory B-cell binding specificities to include recognition of RBD-62. Two weeks following the challenge and initiation of RBD-62 treatment, only 8% of memory B cells in the treatment group with specificities for RBD-62 and/or Delta bound to RBD-62. This was not meaningfully different from the control group (Fig. 5). Further, as a frequency of total memory B cells, the RBD-62-binding population following treatment was hard to distinguish from the pre-challenge background staining (Supplementary Fig. 9c).

## Discussion

As newly emerging SARS-CoV-2 variants continue to evolve while evading prior immunity, acquiring mutations which could render existing anti-viral drugs ineffective and even potentially increasing their affinity for ACE2^2–7,27,28,41–45^, it is essential to develop new treatments for COVID-19 which are variant-agnostic. However, to the best of our knowledge, no host-targeting treatments that are designed specifically to reduce virus load have been tested in clinical trials or within an advanced pre-clinical challenge model such as nonhuman primates. Here we describe the in vivo validation in rhesus macaques of RBD-62, a therapeutic agent which was designed through in vitro evolution to outcompete SARS-CoV-2 RBD for binding to human ACE2 while not interfering with the receptor’s natural enzymatic activity. Targeting the host receptor rather than the virus itself has several benefits including the avoidance of selective pressure and the ability to retain efficacy despite the emergence of new variants, which are unlikely to achieve the 1000-fold increase in ACE2-binding affinity needed to gain a competitive advantage over RBD-62. Moreover, optimizing aerosol delivery of this protein^33^ promotes specific targeting of the respiratory track, including the lungs, achieving a significant reduction in virus replication for the duration of treatment. Importantly, after drug delivery was terminated, virus titers remained lower in the BAL. At the therapeutic dosage used here, RBD-62 did not inhibit the induction of serum or mucosal IgG or IgA responses to the challenge virus or other variants tested. T cell and B-cell immunity were also preserved with no evidence of anti-drug immunity which would have precluded the reuse of this therapeutic agent upon subsequent reinfection.

The benefits of an ACE2 antagonist could theoretically extend to other host-targeting approaches including anti-ACE2 antibodies^46,47^ and ACE2 decoys that bind to RBD^48–58^. It is noteworthy that chiropteran ACE2 functions as a host receptor for NeoCoV and PDF-2180, two merbecoviruses which have so far been restricted to transmission within bats^59^. Continued work on developing biomolecules such as RBD-62 that can block the interaction between SARS-CoV-2 S and human ACE2 has potential benefits not only in this current pandemic but also in our continued vigilance against potential spillover events.

Advancement of this therapeutic agent, or similar host-targeting drugs, into clinical trials would require further optimization of dosage. It is possible that higher doses of RBD-62, or a longer duration of treatment, would have further suppressed virus replication, maintaining a significant protective effect until complete clearance. However, any advantage provided by increasing the amount of RBD-62 would have to be balanced with the possibility of inducing ADA responses.

It has not escaped our notice that the loss of protection following treatment cessation coincided with a delayed primary immune response as indicated by slower kinetics of measurable IgG and IgA titers as well as ACE2-binding inhibitory antibodies and T cell responses. This is likely due to the low levels of virus antigen resulting from RBD-62 treatment, a conclusion supported by a recent publication on the muted primary response arising during nirmatrelvir treatment^60^ and could inform our understanding of the mechanisms contributing to virus rebound following cessation of anti-viral treatment. This would suggest that an increase in the amount of antigen available to elicit an immune response without a commensurate increase in virus replication may be beneficial during RBD-62 treatment. Thus, one approach that may be worth exploring would be vaccination at the time of treatment. Regarding the potential for rebound following RBD-62 treatment, it is notable that the muted primary response did not delay virus clearance as compared to control animals. However, the risk of rebound may be enhanced in cases where treatment is stopped prematurely or if patients are immunocompromised.

Additionally, the protective effect of RBD-62 was striking in that it was observed in both the nose and the lungs. Indeed, our previous findings using mRNA vaccines delivered intramuscularly to nonhuman primates have shown that protection is often either delayed or absent in the upper airway, likely due to the higher threshold of antibodies required for virus suppression in the nose as compared to the lungs^16,34,35,61,62^. Aerosolization of RBD-62 through the PARI nebulizer enables efficient delivery to both the lungs and the nose, highlighting the potential for this medication not only to be administered in a hospital setting to reduce the severity of disease but also to block infection. Indeed, RBD-62 could be employed as a preventative agent for healthcare workers or immunocompromised individuals who cohabitate with an infected individual. In the case of healthcare personnel, preventative treatment could be administered immediately preceding and after attendance to SARS-CoV-2-infected patients when the risk of exposure would be high, mimicking the treatment course described in our study. In the context of widespread immunity from prior infections and the global vaccination campaign, any impact that the use of RBD-62 might have on transmission reduction could accelerate the transition of the current pandemic into an endemic phase.

### Limitations of the study

Although the treatment course that we have designed here may be feasible in a clinical setting, it would have limited applicability to exposure outside of the hospital. Thus, further characterization of RBD-62 as a post-exposure therapeutic agent, as well as modifications to extend protein half-life enabling its utilization as a preventative agent, is warranted. Second, as we have not rechallenged these animals, we cannot definitively determine the impact of the reduction in the magnitude of the primary response on the prevention of reinfection. Nevertheless, it is noteworthy that differences in the immune response between the control and RBD-62-treated NHP are primarily at the level of antibody titers while memory B-cell frequencies and variant specificities are largely preserved. This would suggest that any negative effect on immunity would be primarily at the level of protection against breakthrough infection, rather than severe disease, as anamnestic responses are sufficient to protect the lungs even when circulating antibody titers are suboptimal^35^. Further, it is likely that exposure to SARS-CoV-2 during RBD-62 administration would also occur in the context of multiple previous exposures to vaccine and/or virus, rendering any potential drug-derived impact on immune responses negligible.

The finding that RBD-62 maintains its potency against a panel of different variants stands in stark contrast to the declining efficacy of currently approved vaccines and previously authorized monoclonal antibodies. Indeed, there is no clinically available monoclonal antibody with the capacity to neutralize the currently circulating Omicron sublineages^63–67^. Vaccine development is predicated on predictions of which strains will be dominant at a future time. This can result in both significant lag times between the identification of new strains with transmission advantages and authorization of variant-matched vaccine boosts and also in a mismatch between the vaccine immunogen and the currently circulating variant. As an alternative approach, we have described a rationally designed therapeutic agent which can be used to treat or prevent COVID-19 regardless of future SARS-CoV-2 evolution.

## Methods

### Experimental design

All experiments were conducted according to the National Institutes of Health (NIH) standards on the humane care and use of laboratory animals, and all procedures were approved by and conducted in accordance with regulations of the Animal Care and Use Committees of the NIH Vaccine Research Center (VRC) and BIOQUAL, Inc. (Rockville, MD). Animals were housed and cared for in accordance with local, state, federal and institute policies in facilities accredited by the American Association for Accreditation of Laboratory Animal Care, under standards established in the Animal Welfare Act and the Guide for the Care and Use of Laboratory Animals. Animals were housed in ABSL-2 conditions before challenge. Up to a week prior to (for acclimation) and during the challenge phase of the study, animals were housed in ABSL-3 conditions, per Bioqual facility standard operating procedures.

Four- to seven-year-old male Indian-origin rhesus macaques (*Macaca mulatta*) from a VRC-owned resource colony were sorted into two groups of 8 NHP based on age and weight. RBD-62 formulated in gelatin was administered to one group at the time of Delta challenge while PBS formulated in gelatin was administered to the other group. Animals were challenged with 2 × 10^5^ TCID_50_ of Delta (BEI, NR-56116). 1.5 × 10^5^ TCID_50_ was administered via intratracheal route, and 0.25 × 10^5^ TCID_50_ was administered intranasally to each nostril.

### Production and purification of RBD-62

The RBD-62 protein was produced in several batches to a total of 4.6L Expi293F cells (Thermo Fisher) by transient transfection of the pCAGGS plasmid^32^. Plasmid DNA purified by NucleoBond Xtra Midi kit (Macherey-Nagel) was transfected using ExpiFectamine 293 Transfection Kit (Thermo Fisher) according to the manufacturer’s protocol. Media was collected 72–96 h post transfection, when the cell viability decreased to 50%, by centrifugation (500 × *g* for 15 min). The media was clarified by filtration through a 0.45 µm Nalgene (Thermo Fisher) filter and loaded on two 5 ml HisTrap Fast Flow columns (Cytivia) connected in series using ÄKTA pure system (Cytivia). The column was washed with five column volumes of PBS 20 mM imidazole pH 7.4 and eluted by step elution of 60% elution buffer PBS, 500 mM imidazole pH 7.4. Eluted protein was concentrated by Amicon Ultra Centrifugal Filter Units, MWCO 3 kDa (Merck Millipore Ltd.), and uploaded onto Superdex 200 16/600 (Cytiva) preequilibrated in PBS. The purity and quality of the eluted protein were analyzed by SDS–PAGE and Tycho (Nanotemper), respectively.

### In vitro RBD-62 inhibition of S–ACE2 binding

Inhibitors including RBD-62, WA1 RBD (VRC, NIH) and truncated *Plasmodium falciparum* circumsporozoite protein 5/3_SAmut (Robert Seder, VRC, NIH) were chosen for comparison due to similar molecular weight (~25–27 kDa). Both RBD-62 and WA1 RBD were biotinylated via AviTag. 5/3_Samut^68^ sequence is available on GenBank (ID: MT891178.1). All proteins were diluted to 5 μg/mL and then serially diluted fivefold. ACE2-binding inhibition assay was performed with V-Plex SARS-CoV-2 Panel 23 (ACE2) Kit (MSD) per manufacturer’s instructions. Plates were read on MSD Sector S 600 instrument. All samples run in duplicate and normalized to the average luminescent units measured for each variant without the addition of inhibitor, with the average inhibition at each dilution indicated by the icons. IC_50_ values (ng/mL) were calculated via the [Agonist] vs. normalized response—variable slope equation within the nonlinear regression analysis tool in Prism version 9.3.1. IC_90_ values (ng/mL) were calculated via the [Agonist] vs. response—Find ECanything within the nonlinear regression analysis tool in Prism with bottom and top *y*-values constrained to 0 and 100, respectively. IC_50_ and IC_90_ values not listed for irrelevant malaria protein.

### In vitro RBD-62 inhibition of SARS-CoV-2 infection

#### Cells and viruses

VeroE6-TMPRSS2 cells (VRC, NIH) were generated and cultured as previously described^69^. Cell line was authenticated by characterization of TMPRSS2 via the use of an anti-TMPRSS2 flow antibody. nCoV/USA_WA1/2020 (WA1), closely resembling the original Wuhan strain, was propagated from an infectious SARS-CoV-2 clone as previously described^70^. Omicron BA.1 (EPI_ISL_7171744) was isolated as previously described^3^. Omicron BA.5 isolate (EPI_ISL_13512579) was provided by Dr. Richard Webby (St. Jude Children’s Research Hospital), Omicron XBB.1.5 (EPI_ISL_16026423) was provided by Dr. Andrew Pekosz (Johns Hopkins Bloomberg School of Public Health) and Omicron JN.1 (EPI_ISL_18403077) was provided by Dr. Benjamin Pinsky (Stanford University). All variants were plaque purified and propagated once in VeroE6-TMPRSS2 cells to generate working stocks. Viruses were then deep sequenced and confirmed as previously described^71^.

#### Inhibition assay

To test the anti-viral activity of the RBD proteins, VeroE6-TMPRSS2 cells were seeded in a 96-well plate 1 day before infection. The inhibitors were serially diluted and added to the cells and then incubated for an hour at 37 °C. Inhibitors included RBD-62, WA1 RBD (VRC, NIH) and a resurfaced HIV-1 Env core (RSC3KO) (VRC, NIH). Both RBD-62 and WA1 RBD were biotinylated via AviTag. After 1 h of incubation, cells were infected with various SARS-CoV-2 variants in BSL-3 laboratory and incubated at 37 °C for an additional hour. Post-incubation, the mixture was removed from cells, and 100 μl of prewarmed 0.85% methylcellulose overlay was added to each well. Plates were incubated at 37 °C for 18–40 h (depending on variants). After the appropriate incubation time, the methylcellulose overlay was removed, and cells were washed with PBS and fixed with 2% paraformaldehyde for 30 min. Following fixation, cells were washed twice with PBS, and permeabilized using a permeabilization buffer for at least 20 min. After permeabilization, cells were incubated with an anti-SARS-CoV-2 spike primary antibody directly conjugated to Alexa Fluor-647 (clone CR3022-AF647, Cell Signaling #37475 at a dilution of 1:5000) overnight at 4 °C. Cells were then washed twice with 1× PBS and imaged on an ELISPOT reader (CTL Analyzer). The number of foci for each sample was counted using the Viridot program^72^. Cell viability was determined with compound-treated or mock-treated cells using CellTiter-Glo (Promega), which measures cellular ATP content. All experiments were conducted in quadruplicate, and all values were normalized to mock-treated cells for analysis. Normalized values were used to fit a 4-parameter equation to semi-log plots of the concentration-response data using GraphPad Prism version 10.2.0. IC_50_ and IC_90_ values not listed for irrelevant HIV-1 protein.

### RBD-62 administration

RBD-62 was provided by Gideon Schreiber (Weizmann Institute of Science) and formulated with gelatin as a delivery vehicle. Gelatin (Sigma-Aldrich, G1890) was prepared at a concentration of 4 mg/mL in Dulbecco’s PBS (Gibco). RBD-62 or PBS control was then mixed with gelatin at a 1:1 ratio. Each animal was administered 2.5 mg RBD-62 or PBS control at an effective concentration of 2 mg/mL gelatin with a total volume of 4.6 mL via a pediatric mask attached to a Pari eFlow nebulizer (PARI GmbH) that delivered 4 μm particles deep into the lung of anesthetized macaques, as previously described^73^.

### Subgenomic RNA quantification

sgRNA was isolated and quantified by researchers blinded to vaccine status as previously described^61^. Briefly, total RNA was extracted from BAL fluid and NS using RNAzol BD column kit (Molecular Research Center). PCR reactions were conducted with TaqMan Fast Virus 1-Step Master Mix (Applied Biosystems), forward primer in the 5′ leader region and N gene-specific probe and reverse primer as previously described^16^:

sgLeadSARSCoV2_F: 5′-CGATCTCTTGTAGATCTGTTCTC-3′

N2_P: 5′-FAM-CGATCAAAACAACGTCGGCCCC-BHQ1-3′

wtN_R: 5′-GGTGAACCAAGACGCAGTAT-3′

Amplifications were performed with a QuantStudio 6 Pro Real-Time PCR System (Applied Biosystems). The assay lower LOD was 50 copies/reaction.

### TCID_50_ quantification of SARS-CoV-2 from BAL and NS

TCID_50_ assay was conducted as described previously^61^. Briefly, Vero-TMPRSS2 cells (VRC/NIH) were plated at 25,000 cells/well in Dulbecco’s Modified Eagle Medium (DMEM) + 10% FBS + gentamicin and the cultures were incubated at 37 °C, 5.0% CO_2_. Cells reached 80–100% confluence the following day. The medium was aspirated and replaced with 180 μL of DMEM + 2% FBS + gentamicin. Twenty microliters of BAL or NS sample was added to the top row in quadruplicate and mixed using a P200 pipettor five times. Using the pipettor, 20 μL was transferred to the next row, and repeated down the plate (columns A–H) representing tenfold dilutions. The tips were disposed for each row and repeated until the last row. Positive (virus stock of known infectious titer in the assay) and negative (medium only) control wells were included in each assay set-up. The plates were incubated at 37 °C, 5.0% CO_2_ for 4 days. The cell monolayers were then visually inspected for cytopathic effect. The TCID_50_ value was calculated using the Read–Muench formula.

### Serum and mucosal antibody titers

Quantification of antibodies in the blood and mucosa was performed as previously described^74^. Briefly, total IgG and IgA antigen-specific antibodies to SARS-CoV-2-derived antigens were determined in a multiplex serology assay by Meso Scale Discovery (MSD). We measured responses using V-Plex SARS-CoV-2 Panel 22 for variant RBD and Panel 1 for WT proteins and protein domains according to manufacturer’s instructions, except 25 μl of sample and detection antibody were used per well. BAL and NW were initially concentrated tenfold using Amicon Ultra centrifugal filter 10 kDa MWCO (Millipore). For measurement of antibody titers to variant RBD, concentrated BAL and NW were initially diluted 1:5 and then serially diluted 1:5; heat-inactivated plasma was initially diluted 1:100 and then serially diluted 1:4. Data presented as AUC. For measurement of IgG antibody titers to SARS-CoV-2 protein domains, concentrated BAL and NW were diluted at 1:5, 1:10 and 1:20 ratios; heat-inactivated plasma was diluted at 1:25, 1:50 and 1:100 ratios. For measurement of IgA titers, concentrated BAL and NW were initially diluted 1:5 and then serially diluted 1:5. Results were reported as BAU/mL based upon the reference standard included in Panel 1 kit according to manufacturer’s instructions.

### RBD–ACE2 binding inhibition

BAL fluid and NW were concentrated tenfold with Amicon Ultra centrifugal filter 10 kDa MWCO (Millipore). To remove residual RBD-62 prior to the binding inhibition assay, fluid was diluted 1:1 in 50 mM sodium phosphate, 300 mM sodium chloride (binding buffer). HisPur Ni-NTA spin plate (Thermo Scientific) was equilibrated with binding buffer and the diluted fluid was applied to the plate and incubated with agitation at 4 °C overnight. The purified fluid was collected after centrifugation for 1 min at 1000 × *g*. The fluid was then dialyzed against Diluent 100 using Pierce Microdialysis Plates (Thermo Scientific). Purified fluid was diluted to a final ratio of 1:5. ACE2-binding inhibition assay was performed with V-Plex SARS-CoV-2 Panel 22 (ACE2) Kit (MSD) per manufacturer’s instructions. Plates were read on MSD Sector S 600 instrument. Results are reported as percent inhibition.

### Intracellular cytokine staining (ICS)

Cryopreserved PBMC and BAL cells were thawed and rested overnight in a 37 °C, 5% CO_2_ incubator. The next morning, cells were stimulated with SARS-CoV-2 spike protein (S1 and S2, matched to ancestral COVID-19 mRNA vaccine insert) and nucleocapsid (N) peptide pools (JPT Peptides) at a final concentration of 2 μg/ml in the presence of 3 mM monensin for 6 h. The S1, S2 and N peptide pools are comprised of 158, 157 and 102 individual peptides, respectively, as 15mers overlapping by 11 aa in 100% DMSO. Negative controls received an equal concentration of DMSO instead of peptides (final concentration of 0.5%). ICS was performed as previously described^35,75^. The following monoclonal antibodies were used: (1) CD3 APC-CY7, clone SP34.2, BD Biosciences #557757—Lot #0223215 at a dilution of 1:640; (2) CD4 PE-CY5.5, clone S3.5, Invitrogen #MHCD0418—Lot #2303833 at 1:80; (3) CD8 BV570, clone RPA-T8, Biolegend #301038—Lot #B333843 at 1:80; (4) CD45RA PE-CY5, clone 5H9, BD Biosciences #552888—Lot #8110737 at 1:2500; (5) CCR7 BV650, clone G043H7, Biolegend #353234—Lot #B325079 at 1:10; (6) CXCR5 PE, clone MU5UBEE, Thermo Fisher #12-9185-42—Lot #2279157 at 1:10; (7) CXCR3 BV711, clone 1C6/CXCR3, BD Biosciences #563156—Lot #0309602 at 1:20; (8) PD-1 BUV737, clone EH12.1, BD Horizon #612792—Lot #0303349 at 1:40; (9) ICOS PE-CY7, clone C398.4A, Biolegend #313520—Lot #B213626 at 1:640; (10) CD69 ECD, clone TP1.55.3, Beckman Coulter #6607110—Lot #7620090 at 1:40; (11) IFN-g Alexa 700, clone B27, Biolegend #506516—Lot #B320892 at 1:640; (12) IL-2 BV750, clone MQ1-17H12, BD Biosciences #566361—Lot #7108833 at 1:40; (13) IL-4 BB700, clone MP4-25D2, BD Biosciences custom order—Lot #1042139 at 1:20; (14) TNF FITC, clone Mab11, BD Biosciences #554512—Lot #0015360 at 1:80; (15) IL-13 BV421, clone JES10-5A2, BD Biosciences #563580—Lot #0286560 at 1:20; (16) IL-17A BV605, clone BL168, Biolegend #512326—Lot #B319897 at 1:40; (17) IL-21 Alexa 647, clone 3A3-N2.1, BD Biosciences #560493—Lot #1005849 at 1:10; and (18) CD154 BV785, clone 24-31, Biolegend #310842—Lot #B329207 at 1:20. Aqua Live/Dead Fixable Dead Cell Stain Kit (Invitrogen #L34957—Lot #2204200 at 1:800) was used to exclude dead cells. All antibodies were previously titrated to determine the optimal concentration. Samples were acquired on a BD FACSymphony flow cytometer and analyzed using FlowJo version 10.8.2 (Treestar, Inc., Ashland, OR).

### B-cell probe binding

Flow cytometric analysis of antigen-specific memory B-cell frequencies was performed as previously described^35^. Briefly, cryopreserved PBMC were thawed and stained with the following antibodies (monoclonal unless indicated): (1) IgD FITC, goat polyclonal antibody, Southern Biotech #2030-02—Lot #A2118-WF09C at a dilution of 1:40; (2) IgM PerCP-Cy5.5, clone G20-127, BD Biosciences #561285—Lot #0307134 at 1:40; (3) IgA Dy405, goat polyclonal antibody, Jackson ImmunoResearch #109-475-011—Lot #155196 at 1:40; (4) CD20 BV570, clone 2H7, Biolegend #302332—Lot #B301458 at 1:40; (5) CD27 BV650, clone O323, Biolegend #302828—Lot #B273921 at 1:20; (6) CD14 BV785, clone M5E2, Biolegend #301840—Lot #B327948 at 1:80; (7) CD16 BUV496, clone 3G8, BD Biosciences #564653—Lot #0288806 at 1:40; (8) CD4 BUV737, clone SK3, BD Biosciences #564305—Lot #0282762 at 1:40; (9) CD8 BUV395, clone RPA-T8, BD Biosciences #563795—Lot #9346411 at 1:80; (10) CD19 APC, clone J3-119, Beckman Coulter #IM2470U—Lot #200092 at 1:20; (11) IgG Alexa 700, clone G18-145, BD Biosciences #561296—Lot #0135021 at 1:20; (12) CD3 APC-Cy7, clone SP34.2, BD Biosciences #557757—Lot #0223215 at 1:40; (13) CD38 PE, clone OKT10, Caprico Biotech #100826—Lot #8AE4 at 1:640; (14) CD21 PE-Cy5, clone B-ly4, BD Biosciences #551064—Lot #0072939 at 1:20; and (15) CXCR5 PE-Cy7, clone MU5UBEE, Thermo Fisher #25-9185-42—Lot #2312036 at 1:40. Stained cells were then incubated with streptavidin-BV605 (BD Biosciences) labeled Delta S-2P, BA1 S-2P or RBD-62 and streptavidin-BUV661 (BD Biosciences) labeled WA1 or Delta S-2P for 30 min at 4 °C (protected from light). Cells were washed and fixed in 0.5% formaldehyde (Tousimis Research Corp) prior to data acquisition. Aqua Live/Dead Fixable Dead Cell Stain Kit (Invitrogen #L34957—Lot #2098529 at 1:800) was used to exclude dead cells. All antibodies were previously titrated to determine the optimal concentration. Samples were acquired on a BD FACSymphony cytometer and analyzed using FlowJo version 10.7.2 (BD, Ashland, OR).

### Quantification and statistical analysis

Comparisons of animals that received RBD-62 vs. control for virus titers and humoral responses post-challenge are based on Wilcoxon tests on individual days while longitudinal analyses are based on generalized estimating equations (GEE). We adjusted for multiple comparisons across timepoints for each assay using Holm’s adjustment; we did not adjust to account for multiple comparisons across different assays or different target antigens. *P* values are shown in the figures, and the relevant statistical analyses and sample *n* are listed in corresponding figure legends. NS denotes that the indicated comparison was not significant, with two-sided *P* > 0.05.

Virus titers were analyzed on the log_10_ scale; humoral responses were analyzed as the AUC on the log_10_ scale. Values below the limit of detection or lower limit of quantification for virus titers were set to half of the value for statistical analysis (25 copies sgRNA or 1.35 TCID_50_ per mL or per swab). Antibody binding and virus assays are log-transformed as appropriate. All statistical analyses were done using R version 4.2.1. All flow cytometry data were graphed in FlowJo version 10.7.2 (B-cell binding) or version 10.8.2 (ICS) while all other graphs were designed using Prism version 9.3.1 or 10.2.0.

### Reporting summary

Further information on research design is available in the Nature Portfolio Reporting Summary linked to this article.

### Supplementary information

Supplementary Information

Reporting Summary

### Source data

Source Data

## Data Availability

All data are available in the main text, the Supplementary data or as Source data provided with this paper. Source data are provided with this paper.
