# Supplementary material for: Variant-proof high affinity ACE2 antagonist limits SARS-CoV-2 replication in upper and lower airways
Source: Nat Commun. 2024 Aug 12;15:6894. doi: 10.1038/s41467-024-51046-w (PMC11319446; doi:10.1038/s41467-024-51046-w)
Supplement: Supplementary file 1 — Supplementary Information [file 41467_2024_51046_MOESM1_ESM.pdf]

Supplementary data for

# Variant-proof high affinity ACE2 antagonist limits SARS-CoV-2 replication in upper and lower airways

Matthew Gagne, Barbara J. Flynn, Christopher Cole Honeycutt, Dillon R. Flebbe, Shayne F. Andrew, Samantha J. Provost, Lauren McCormick, Alex Van Ry, Elizabeth McCarthy, John-Paul M. Todd, Saran Bao, I-Ting Teng, Shir Marciano, Yinon Rudich, Chunlin Li, Shilpi Jain, Bushra Wali, Laurent Pessaint, Alan Dodson, Anthony Cook, Mark G. Lewis, Hanne Andersen, Jiří Zahradník, Mehul S. Suthar, Martha C. Nason, Kathryn E. Foulds, Peter D. Kwong, Mario Roederer, Gideon Schreiber, Robert A. Seder*, and Daniel C. Douek*

*Corresponding authors. Email: ddouek@mail.nih.gov (DCD); rseder@mail.nih.gov (RAS)

| RBD-62 Sequence |
| --- |
| TNLCPFGEVFNATRFASVYAWNRKRFSNCVADYSVLYNSASFSTFKCYGVSPTKLND LCFTNVYADSFVIRGDEVRQIAPGQTGKIADYNYKLPDDFTGCVIAWNSNNLDSKKG GNYNYLYRLFRKSKLKPFERDTSMEIYQAGNTPCNGVKGFNCYFPLQSYGFRPTYGV GYQPYRVVVLSFELLHAPATVCGPKHHHHHH |

**Supplementary Table 1.**

**RBD-62 Sequence.** Amino acid sequence of His-tagged RBD-62.

| sgRNA_N | | | | | |
|---|---|---|---|---|---|
| Sample Type | Day Post-challenge | *P* Value (Wilcoxon) | Estimated Reduction in Copies sgRNA ($Log_{10}$) | 95% Confidence Interval Range of Reduction ($Log_{10}$) | |
| BAL | 2 | 0.0031 | 2.5178 | 1.0834 | 3.6414 |
| | 4 | 0.0416 | 1.0698 | 0.5664 | 2.2385 |
| | 7 | NS (0.1660) | 1.0137 | -0.2814 | 1.7335 |
| | 9 | NS (0.1660) | 1.1653 | -0.4116 | 2.1651 |
| | 14 | NS (0.1036) | 0.548 | -0.0001 | 1.5124 |
| NS | 2 | 0.0093 | 1.638 | 0.5856 | 3.825 |
| | 4 | NS (0.1995) | 0.9923 | 0.0633 | 3.1553 |
| | 7 | NS (0.2490) | 0.5582 | -0.0931 | 1.2478 |
| | 9 | NS (0.5573) | -0.7711 | -2.2032 | 1.2688 |
| | 14 | NS (0.6742) | -0.1631 | -1.4874 | 0.6775 |

**Supplementary Table 2.**

**Reduction in virus replication due to RBD-62 treatment.** Table lists *P* values and lower and upper bounds of 95% confidence interval (CI) for differences between RBD-62-treated and control groups ($n$=8) on each indicated day using two-sided Wilcoxon rank-sum test after a Holm's adjustment across timepoints. Data is related to measurement of virus replication in Fig. 2a.

| Virus Titers | | | | | |
|---|---|---|---|---|---|
| Sample Type | Day Post-challenge | *P* Value (Wilcoxon) | Estimated Reduction in $TCID_{50}$ ($Log_{10}$) | 95% Confidence Interval Range of Reduction ($Log_{10}$) | |
| BAL | 2 | 0.0026 | 2.438 | 0.83 | 4.68 |
| | 4 | 0.0411 | 1.17 | 0.0001 | 1.9999 |
| NS | 2 | 0.0404 | 1.5 | 0.33 | 2.9999 |
| | 4 | 0.0439 | 1.17 | <0.0001 | 4.8499 |

**Supplementary Table 3.**

**Reduction in culturable virus due to RBD-62 treatment.** Table lists *P* values and lower and upper bounds of 95% confidence interval (CI) for differences between RBD-62-treated and control groups (*n*=8) on each indicated day using two-sided Wilcoxon rank-sum test after a Holm's adjustment across timepoints. Data is related to measurement of culturable virus in Fig. 2b.

| IgG Binding Titers | | | | | |
|---|---|---|---|---|---|
| Sample Type | Antigen | *P* Value (GEE) | Estimated Reduction in AUC ($Log_{10}$) | 95% Confidence Interval Range of Reduction ($Log_{10}$) | |
| Serum | WT | NS (0.1261) | 0.6094 | -0.1715 | 1.3902 |
| | Delta | 0.0132 | 0.8967 | 0.1876 | 1.6057 |
| | BA.1 | NS (0.4720) | 0.2261 | -0.3901 | 0.8424 |
| BAL | WT | <0.0001 | 1.7599 | 0.9830 | 2.5369 |
| | Delta | 0.0001 | 1.5237 | 0.7418 | 2.3056 |
| | BA.1 | 0.0140 | 0.6220 | 0.1258 | 1.1183 |
| NS | WT | 0.0246 | 0.6023 | 0.0769 | 1.1277 |
| | Delta | 0.0044 | 0.6775 | 0.2108 | 1.1442 |
| | BA.1 | NS (0.9125) | 0.0180 | -0.3034 | 0.3394 |

**Supplementary Table 4.**

**Reduction in post-challenge primary responses due to RBD-62 treatment.** Table lists *P* values and lower and upper bounds of 95% CI for treatment effect between RBD-62-treated and control groups (*n*=8) across all post-challenge timepoints using two-sided generalized estimating equations. No adjustments were made for multiple comparisons. Data is related to measurement of post-challenge anti-SARS-CoV-2 RBD binding titers in Fig. 3.

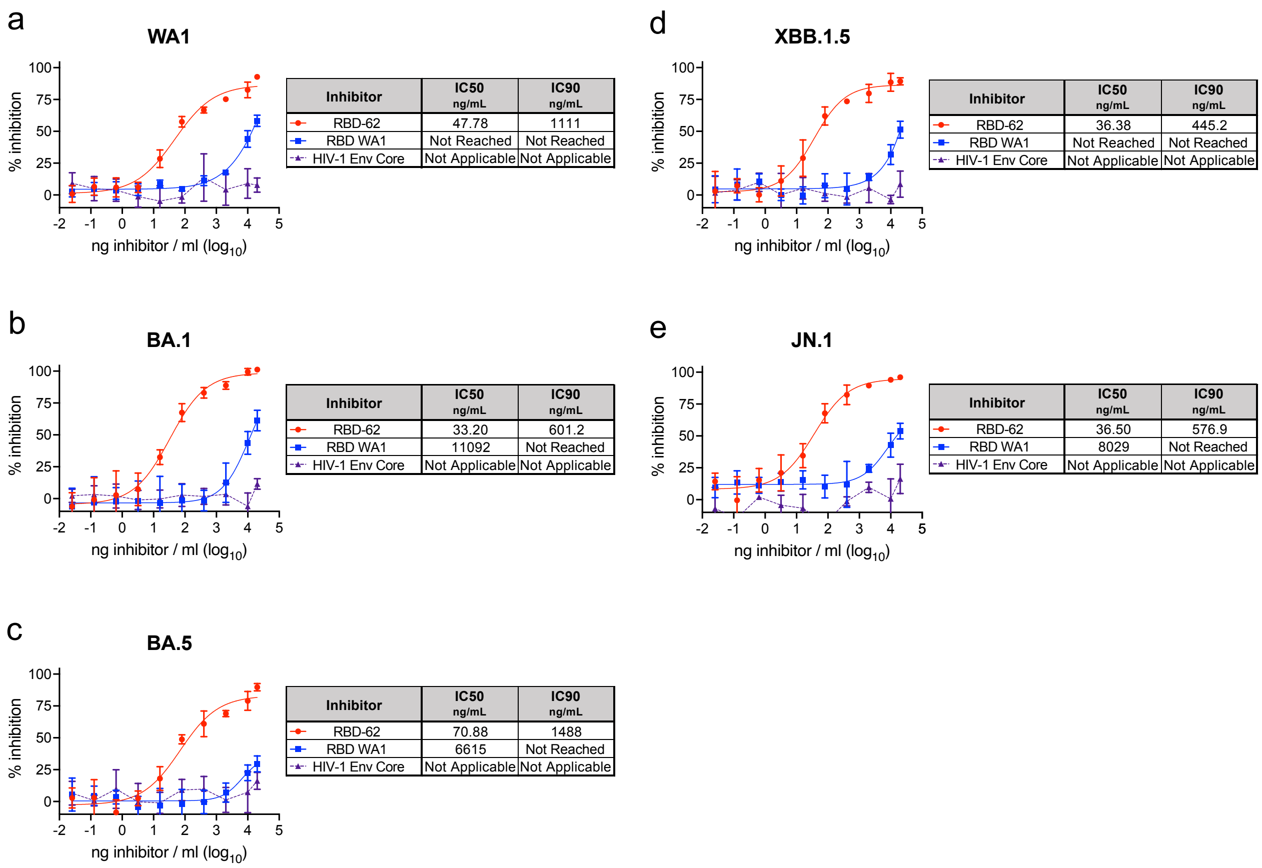


a — WA1

| Inhibitor | IC50 ng/mL | IC90 ng/mL |
|---|---|---|
| RBD-62 | 47.78 | 1111 |
| RBD WA1 | Not Reached | Not Reached |
| HIV-1 Env Core | Not Applicable | Not Applicable |

b — BA.1

| Inhibitor | IC50 ng/mL | IC90 ng/mL |
|---|---|---|
| RBD-62 | 33.20 | 601.2 |
| RBD WA1 | 11092 | Not Reached |
| HIV-1 Env Core | Not Applicable | Not Applicable |

c — BA.5

| Inhibitor | IC50 ng/mL | IC90 ng/mL |
|---|---|---|
| RBD-62 | 70.88 | 1488 |
| RBD WA1 | 6615 | Not Reached |
| HIV-1 Env Core | Not Applicable | Not Applicable |

d — XBB.1.5

| Inhibitor | IC50 ng/mL | IC90 ng/mL |
|---|---|---|
| RBD-62 | 36.38 | 445.2 |
| RBD WA1 | Not Reached | Not Reached |
| HIV-1 Env Core | Not Applicable | Not Applicable |

e — JN.1

| Inhibitor | IC50 ng/mL | IC90 ng/mL |
|---|---|---|
| RBD-62 | 36.50 | 576.9 |
| RBD WA1 | 8029 | Not Reached |
| HIV-1 Env Core | Not Applicable | Not Applicable |

**Supplementary Fig. 1. Inhibition of *in vitro* infection with authentic virus.** VeroE6-TMPRSS2 cells were infected with SARS-CoV-2 S WA1 (**a**), BA.1 (**b**), BA.5 (**c**), XBB.1.5 (**d**) and JN.1 (**e**). Focus-forming unit assay was conducted with a fluorescently-labeled anti-SARS-CoV-2 S primary antibody. Indicated concentrations of RBD-62, RBD from WA1 or an irrelevant resurfaced HIV-1 Env core (RSC3KO) were added at the time of infection to determine percentage infection inhibition. Icons represent average inhibition of quadruplicate technical replicates at each indicated dilution while error bars represent standard deviation. $IC_{50}$ and $IC_{90}$ values (ng/mL) are indicated to the right of each graph and, along with the curves indicated on the graphs, were calculated using the 4-parameter nonlinear regression analysis tool in Prism. Dotted lines indicate background inhibition observed for HIV-1 RSC3KO and may extend beyond the lower range of the y-axis. Source data are provided as a Source Data file.

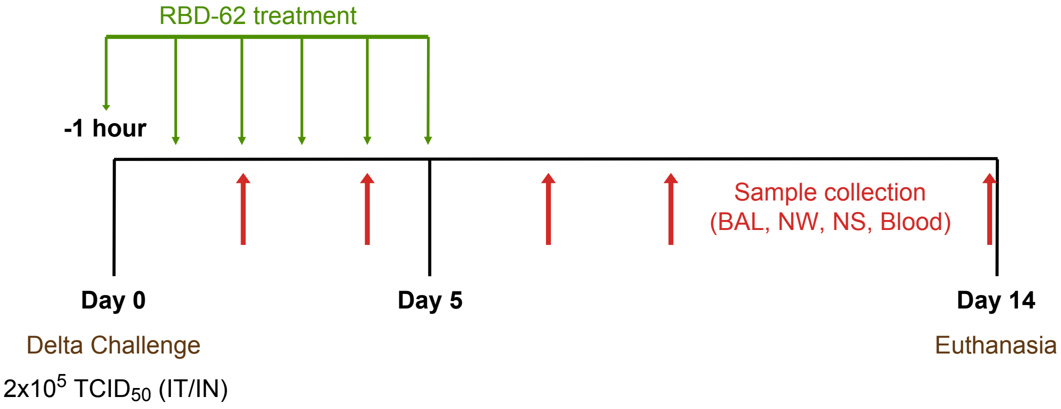


**Supplementary Fig. 2. Experimental schema.** NHP (*n*=8 per group) were challenged with $2x10^5$ $TCID_{50}$ Delta on day 0. RBD-62 was administered to one group of NHP on day 0 (1 hour prior to challenge) and on each successive day up to and including day 5. BAL, NW, NS and blood (both serum and PBMC) were collected at timepoints indicated by red arrows. All animals were euthanized on day 14.

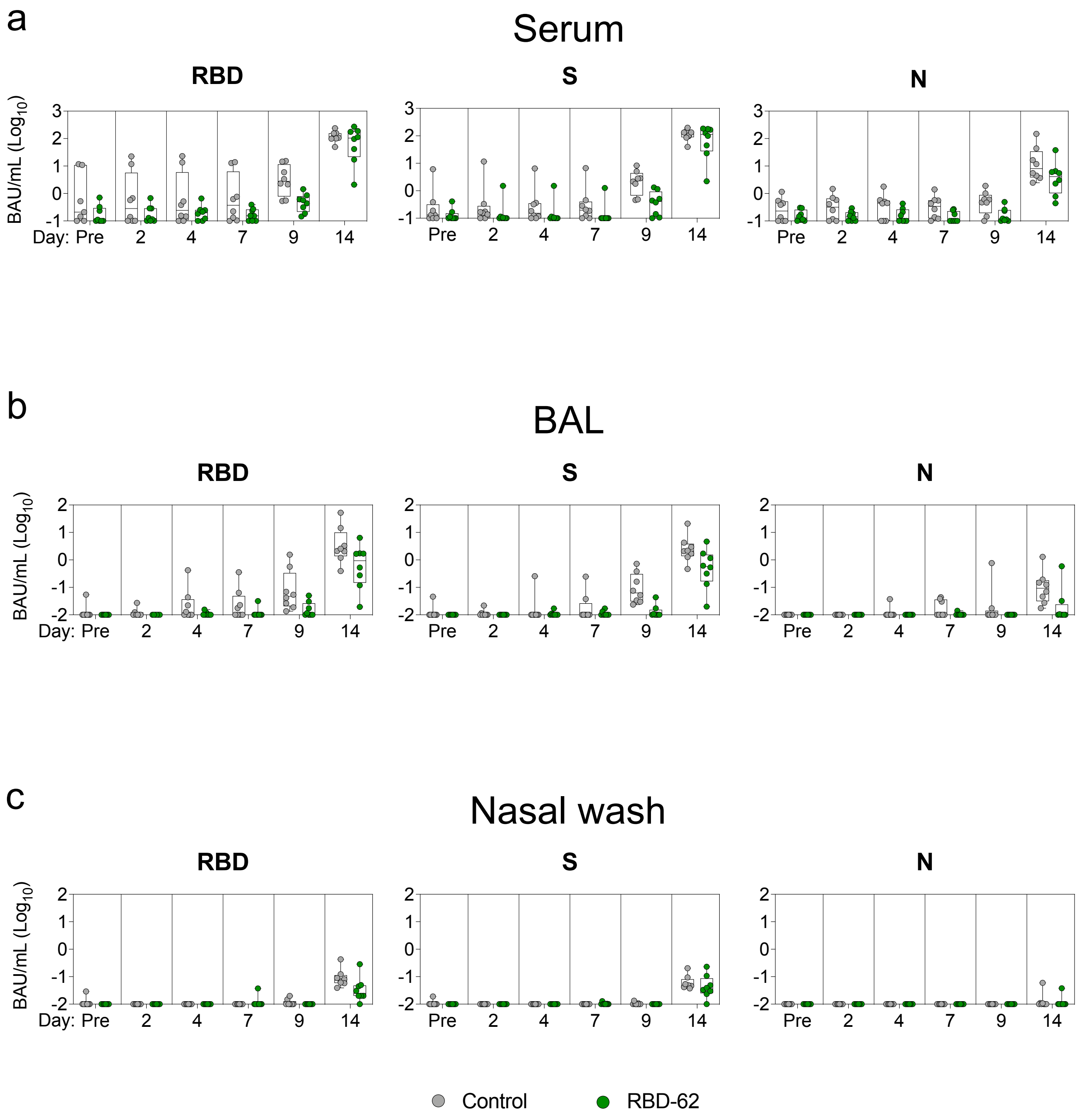


**Supplementary Fig. 3. Humoral responses to SARS-CoV-2 proteins and domains.** NHP (*n*=8 per group) were challenged with $2x10^5$ $TCID_{50}$ Delta and simultaneously treated with RBD-62 (green circles) or PBS (gray circles). IgG binding titers were measured to wildtype RBD, S and N in the (**a**) serum, (**b**) BAL and (**c**) nasal wash one month prior to challenge (pre-challenge) and on days 2, 4, 7, 9 and 14 post-challenge. Binding titers in WHO-specified international units may extend beyond the lower range of the y-axis. Sera were diluted at ratios of 1:25, 1:50 and 1:100 while mucosal fluids were diluted at ratios of 1:5, 1:10 and 1:20. Circles, boxes and horizontal lines represent individual animals, interquartile range and median, respectively, while minima and maxima are denoted at whisker termini. Source data are provided as a Source Data file.

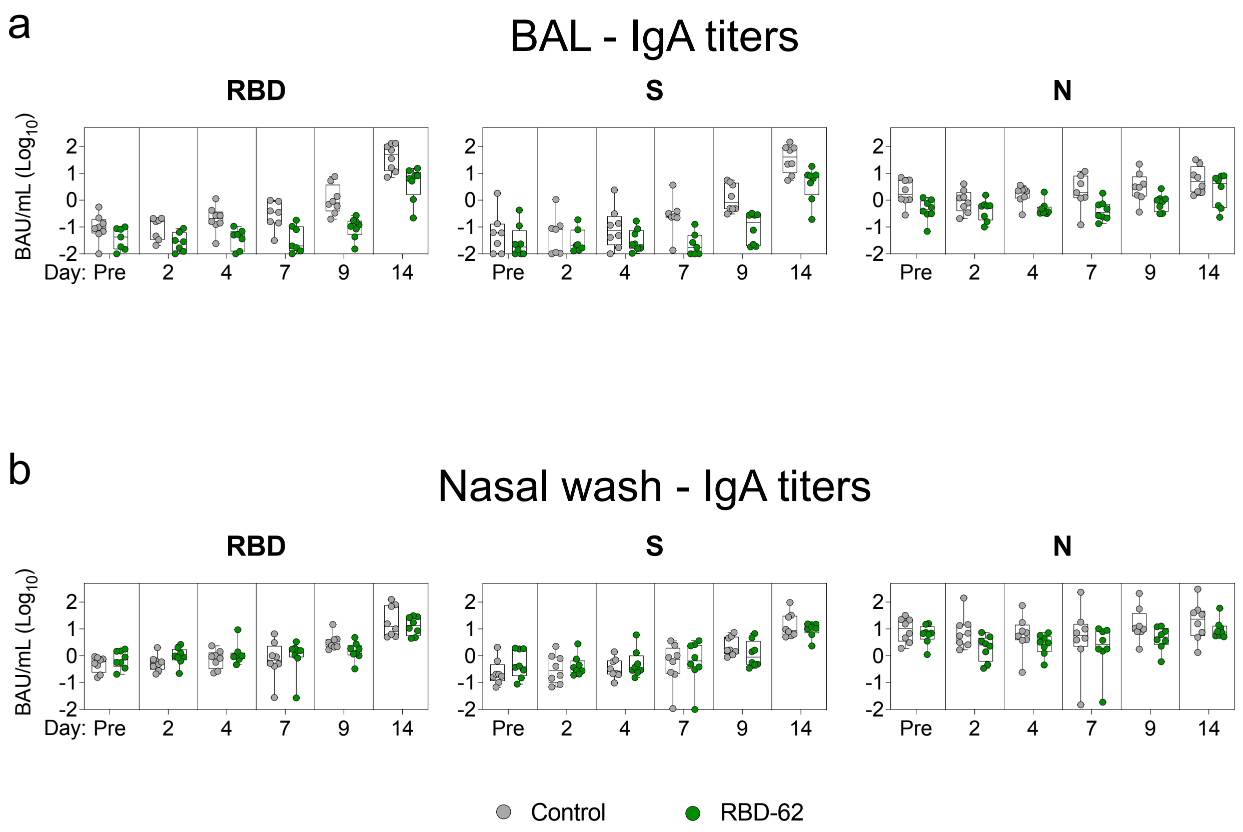


**Supplementary Fig. 4. Mucosal antibody responses to SARS-CoV-2 proteins and domains.** NHP (*n*=8 per group) were challenged with 2x10$^5$ $TCID_{50}$ Delta and simultaneously treated with RBD-62 (green circles) or PBS (gray circles). IgA binding titers were measured to wildtype RBD, S and N in the (**a**) BAL and (**b**) nasal wash one month prior to challenge (pre-challenge) and on days 2, 4, 7, 9 and 14 post-challenge. Binding titers in WHO-specified international units may extend beyond the lower range of the y-axis. Mucosal fluids were initially diluted 1:5 and then serially diluted 1:5. Circles, boxes and horizontal lines represent individual animals, interquartile range and median, respectively, while minima and maxima are denoted at whisker termini. Source data are provided as a Source Data file.

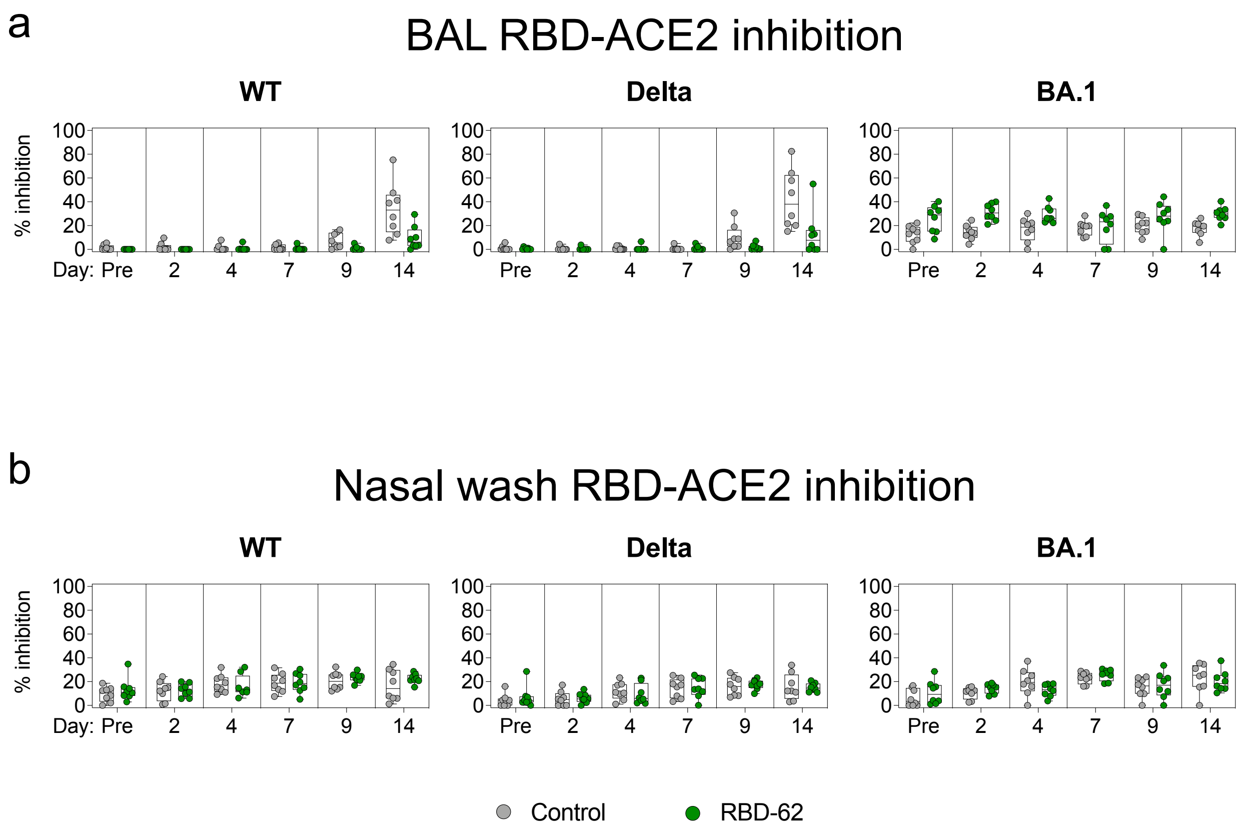


**Supplementary Fig. 5. RBD-ACE2 binding inhibition in the mucosa.** NHP (*n*=8 per group) were challenged with $2x10^5$ $TCID_{50}$ Delta and simultaneously treated with RBD-62 (green circles) or PBS (gray circles). RBD from WT, Delta and BA.1 variants were mixed with soluble ACE2 in combination with mucosal antibodies from the (**a**) BAL or (**b**) nasal wash one month prior to challenge (pre-challenge) and on days 2, 4, 7, 9 and 14 post-challenge. Percentage binding inhibition was determined relative to maximum binding without inclusion of mucosal fluid. All samples were diluted 1:5. Circles, boxes and horizontal lines represent individual animals, interquartile range and median, respectively, while minima and maxima are denoted at whisker termini. Source data are provided as a Source Data file.

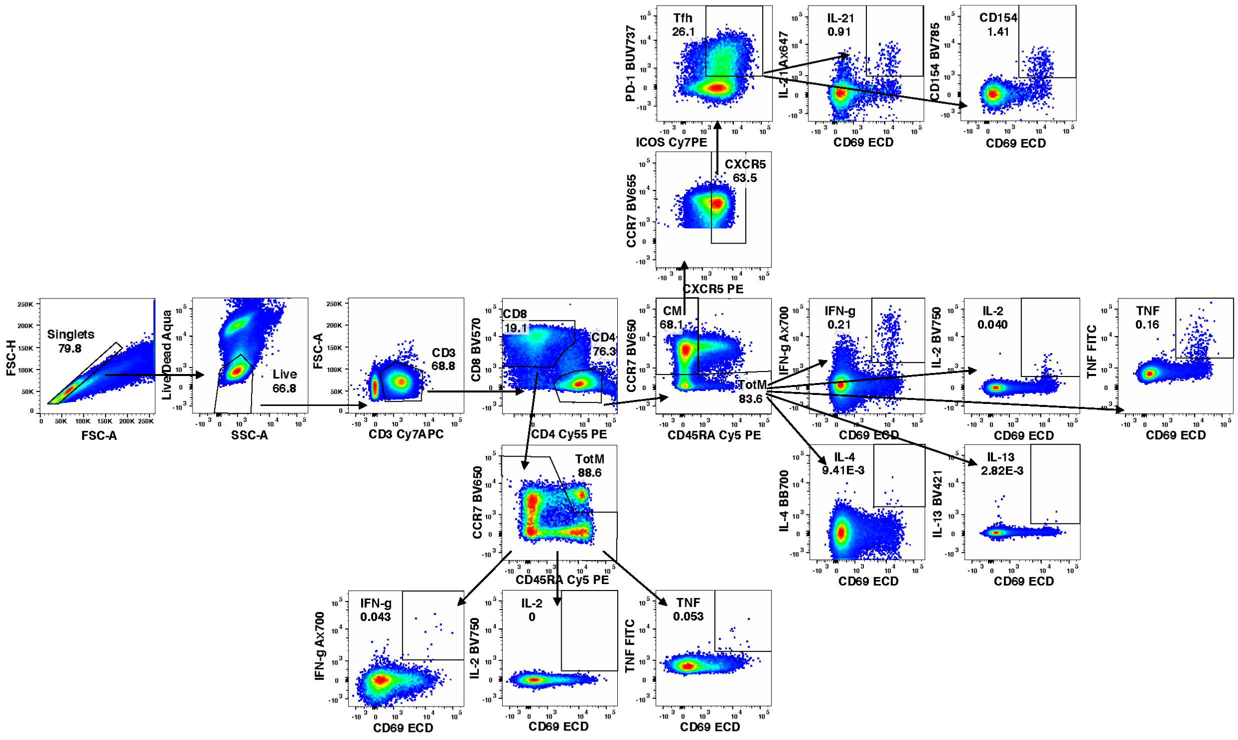


**Supplementary Fig. 6. ICS gating strategy.** Cells were gated as singlets and live cells on forward and side scatter and a live/dead aqua blue stain. $CD3^+$ events were gated as $CD4^+$ or $CD8^+$ T cells. Total memory $CD8^+$ T cells were selected based on expression of CCR7 and CD45RA. Finally, SARS-CoV-2 S-specific memory $CD8^+$ T cells (as shown in Fig. 4c, Fig. 4h and Supplementary Fig. 7c) were gated according to co-expression of CD69 and IL-2, TNF or IFNγ. The $CD4^+$ events were defined as total memory or central memory according to expression of CCR7 and CD45RA. $CD4^+$ cells with a $T_H1$ phenotype (as shown in Fig. 4a, Fig. 4f and Supplementary Fig. 7a) were defined as memory cells that co-expressed CD69 and IL-2, TNF or IFNγ. $CD4^+$ cells with a $T_H2$ phenotype (as shown in Fig. 4b, Fig. 4g and Supplementary Fig. 7b) were defined as memory cells that co-expressed CD69 and IL-4 or IL-13. $T_{FH}$ cells were defined as central memory $CD4^+$ T cells that expressed CXCR5, ICOS and PD-1. $T_{FH}$ cells were further characterized as $IL\text{-}21^+$, $CD69^+$ (as shown in Fig. 4d and Supplementary Fig. 7d) or $CD40L^+$, $CD69^+$ (as shown in Fig. 4e and Supplementary Fig. 7e).

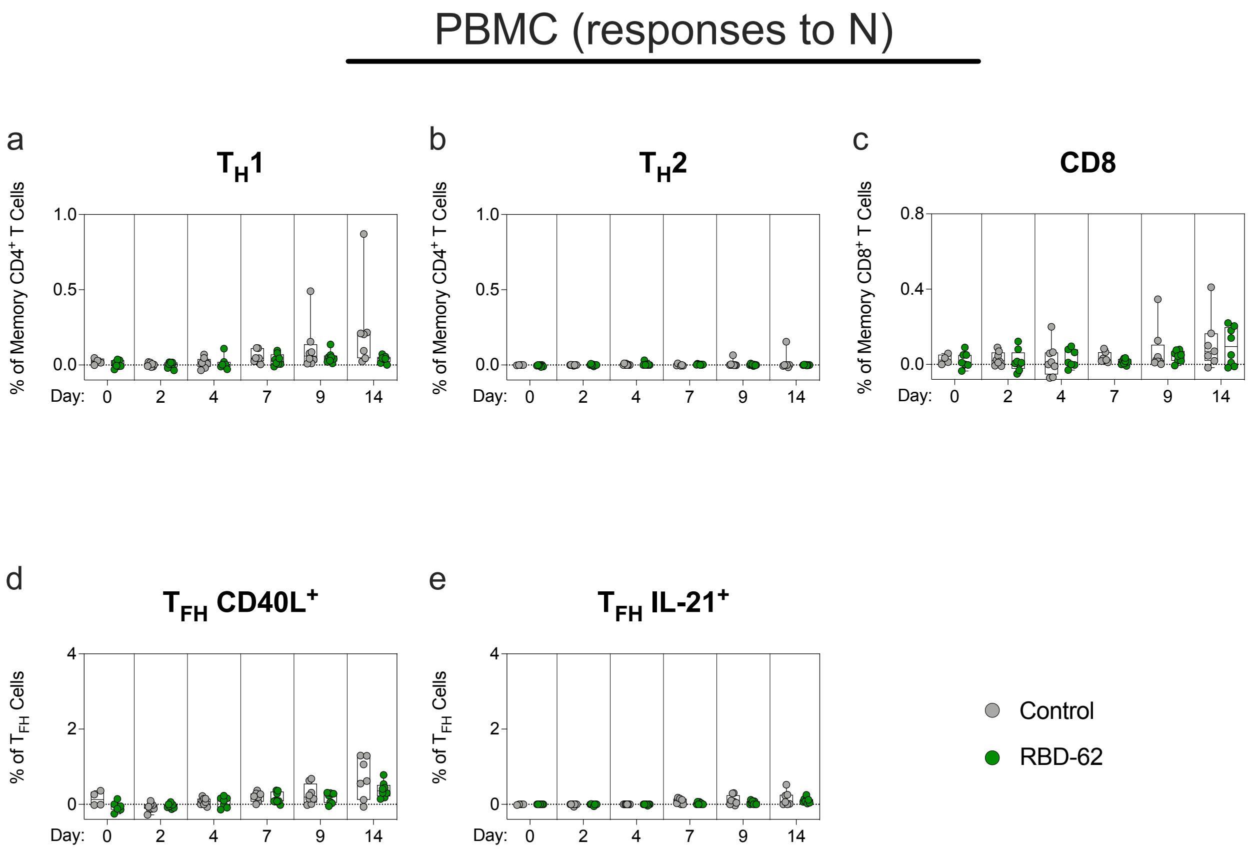


**Supplementary Fig. 7. Kinetics of primary T cell responses to N peptides following Delta challenge.** NHP (*n*=8 per group) were challenged with $2x10^5$ $TCID_{50}$ Delta and simultaneously treated with RBD-62 (green circles) or PBS (gray circles). Peripheral blood mononuclear cells (PBMC) were collected immediately prior to challenge and on days 2, 4, 7, 9 and 14 post-challenge. Cells were stimulated with WA1 N peptide pools and responses measured by intracellular cytokine staining (ICS). (**a**) Percentage of memory $CD4^+$ T cells expressing $T_H1$ markers (IL-2, TNF or IFNγ). (**b**) Percentage of memory $CD4^+$ T cells expressing $T_H2$ markers (IL-4 or IL-13). (**c**) Percentage of memory $CD8^+$ T cells expressing IL-2, TNF or IFNγ. (**d**,**e**) Percentage of $T_{FH}$ cells expressing CD40L or IL-21, respectively. Dotted lines set at 0%. Reported percentages may be negative due to background subtraction. Circles, boxes and horizontal lines represent individual animals, interquartile range and median, respectively, while minima and maxima are denoted at whisker termini. Due to pre-specified minimum cell numbers per sample required for analysis, some timepoints include data from less than 8 NHP per group. Source data are provided as a Source Data file.

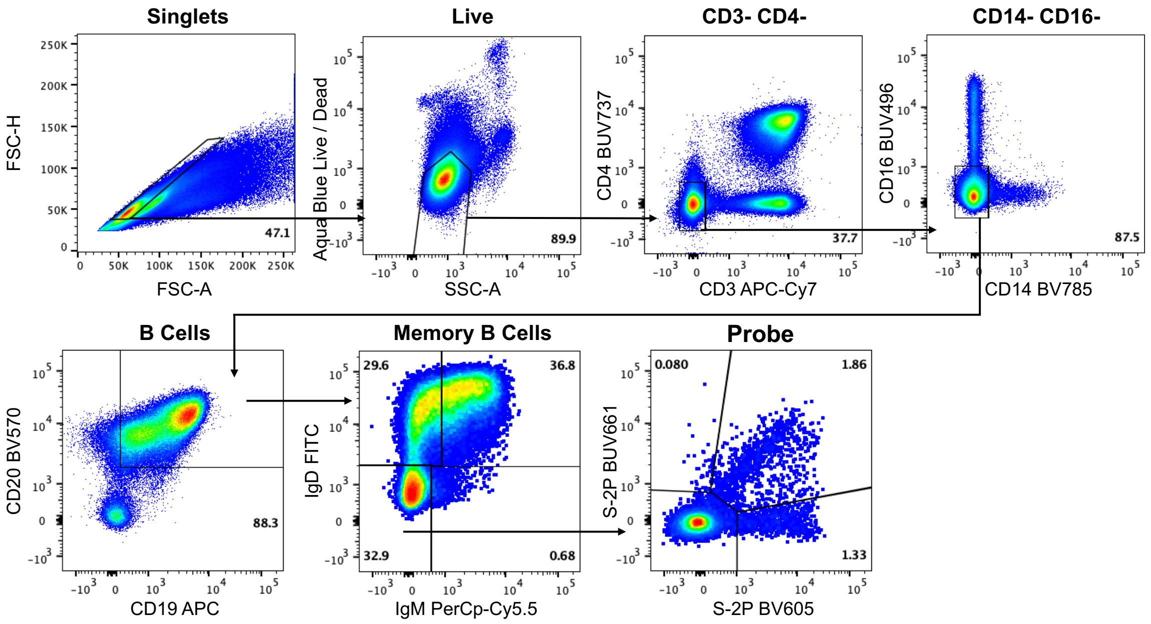


**Supplementary Fig. 8. Memory B cell gating strategy.** Cells were gated as singlets and live cells on forward and side scatter and a live/dead aqua blue stain. Cells were further gated based on lack of expression of CD3, CD4, CD14 and CD16. B cells were then defined based on expression of CD20 and CD19 whereas memory B cells were gated based on lack of IgD or IgM expression. Finally variant S-2P probe pairs were used to define binding specificity. The final panel corresponds to the data shown in Fig. 5 and Supplementary Fig. 9.

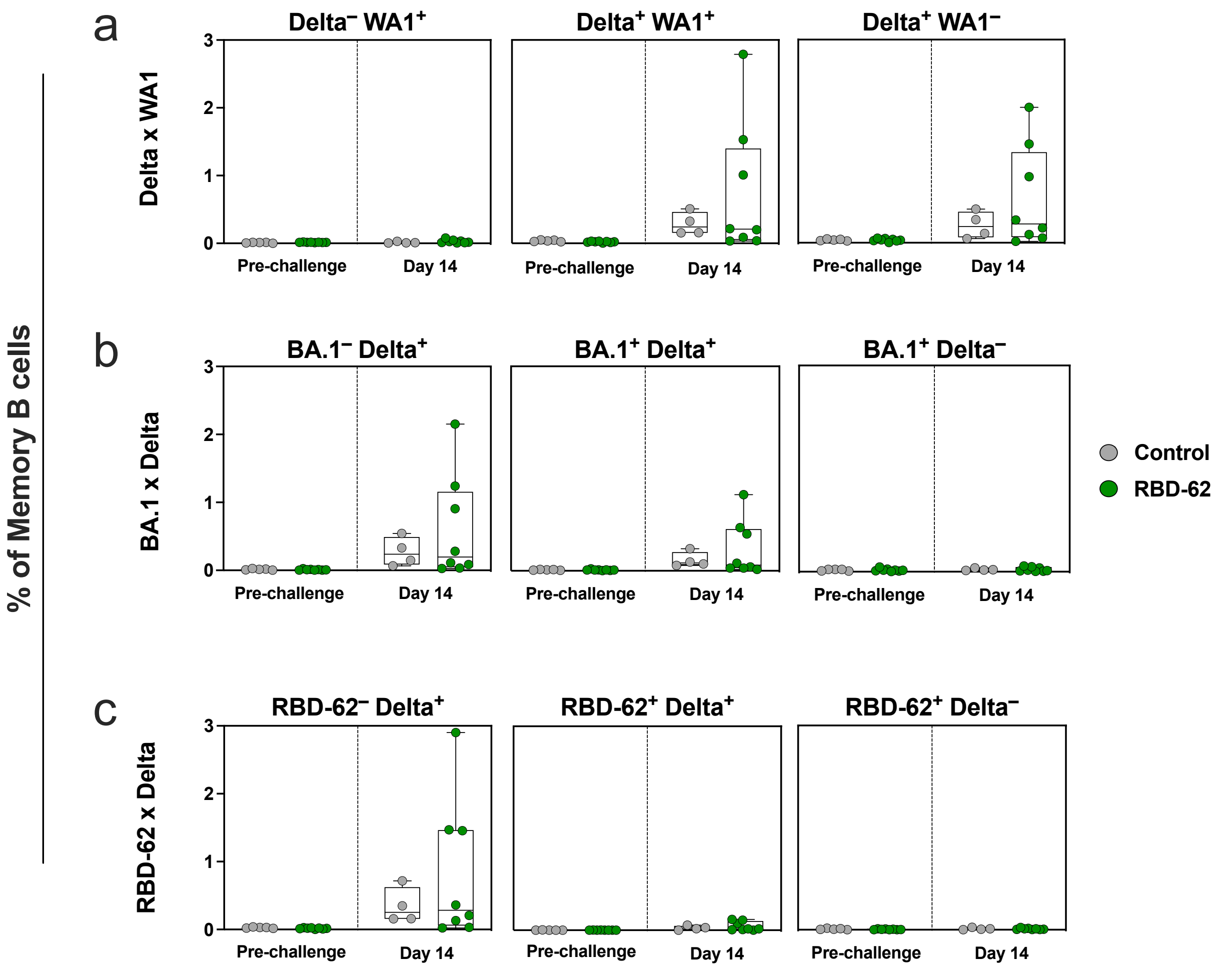


**Supplementary Fig. 9. Variant-specific and cross-reactive memory B cell frequencies.** NHP were challenged with 2x10$^5$ $TCID_{50}$ Delta and simultaneously treated with RBD-62 (green circles) or PBS (gray circles). Memory B cells were collected from the periphery and measured via flow cytometry for binding specificity using pairs of fluorescently-labeled variant probes at day 0 and 14 post-challenge. Probe pairs included (**a**) Delta and WA1 S-2P, (**b**) BA.1 and Delta S-2P and (**c**) RBD-62 and Delta S-2P. Cross-reactive cells shown in middle column while variant-specific memory B cells are displayed in left and right columns. Antigen-specific cells reported as a frequency of total memory B cell population. Circles, boxes and horizontal lines represent individual animals, interquartile range and median, respectively, while minima and maxima are denoted at whisker termini. *n*=8 for treated group and *n*=5 and 4 for control group at pre- and post-challenge timepoints, respectively. Source data are provided as a Source Data file.
