# Supplementary material for: Variant-proof high affinity ACE2 antagonist limits SARS-CoV-2 replication in upper and lower airways
Source: Nat Commun. 2024 Aug 12;15:6894. doi: 10.1038/s41467-024-51046-w (PMC11319446; doi:10.1038/s41467-024-51046-w)
Supplement: Supplementary file 2 — Reporting Summary [file 41467_2024_51046_MOESM2_ESM.pdf]

nature portfolio

Corresponding author(s): Daniel C. Douek, Robert A. Seder

Last updated by author(s): Jul 2, 2024

# Reporting Summary

Nature Portfolio wishes to improve the reproducibility of the work that we publish. This form provides structure for consistency and transparency in reporting. For further information on Nature Portfolio policies, see our Editorial Policies and the Editorial Policy Checklist.

## Statistics

For all statistical analyses, confirm that the following items are present in the figure legend, table legend, main text, or Methods section.

| n/a | Confirmed | |
|---|---|---|
| ☐ | ☒ | The exact sample size (*n*) for each experimental group/condition, given as a discrete number and unit of measurement |
| ☐ | ☒ | A statement on whether measurements were taken from distinct samples or whether the same sample was measured repeatedly |
| ☐ | ☒ | The statistical test(s) used AND whether they are one- or two-sided<br>*Only common tests should be described solely by name; describe more complex techniques in the Methods section.* |
| ☐ | ☒ | A description of all covariates tested |
| ☐ | ☒ | A description of any assumptions or corrections, such as tests of normality and adjustment for multiple comparisons |
| ☐ | ☒ | A full description of the statistical parameters including central tendency (e.g. means) or other basic estimates (e.g. regression coefficient) AND variation (e.g. standard deviation) or associated estimates of uncertainty (e.g. confidence intervals) |
| ☐ | ☒ | For null hypothesis testing, the test statistic (e.g. *F*, *t*, *r*) with confidence intervals, effect sizes, degrees of freedom and *P* value noted<br>*Give P values as exact values whenever suitable.* |
| ☒ | ☐ | For Bayesian analysis, information on the choice of priors and Markov chain Monte Carlo settings |
| ☒ | ☐ | For hierarchical and complex designs, identification of the appropriate level for tests and full reporting of outcomes |
| ☒ | ☐ | Estimates of effect sizes (e.g. Cohen's *d*, Pearson's *r*), indicating how they were calculated |

*Our web collection on statistics for biologists contains articles on many of the points above.*

## Software and code

Policy information about availability of computer code

| | |
|---|---|
| Data collection | All flow cytometry data was collected in BD FACS Diva. |
| Data analysis | All statistical analyses were done using R version 4.2.1. Determination of RBD-62 IC50 values were done using Prism version 9.3.1 (for inhibition of ACE2 binding) or version 10.2.0 (for inhibition of in vitro infection). All flow cytometry data analyzed in FlowJo version 10.7.2 (B cell binding) or version 10.8.2 (ICS). |

For manuscripts utilizing custom algorithms or software that are central to the research but not yet described in published literature, software must be made available to editors and reviewers. We strongly encourage code deposition in a community repository (e.g. GitHub). See the Nature Portfolio guidelines for submitting code & software for further information.

## Data

Policy information about availability of data

All manuscripts must include a data availability statement. This statement should provide the following information, where applicable:

- Accession codes, unique identifiers, or web links for publicly available datasets
- A description of any restrictions on data availability
- For clinical datasets or third party data, please ensure that the statement adheres to our policy

All data are available in the main text, the supplementary data or as source data provided with this paper.

# Research involving human participants, their data, or biological material

Policy information about studies with human participants or human data. See also policy information about sex, gender (identity/presentation), and sexual orientation and race, ethnicity and racism.

| | |
|---|---|
| Reporting on sex and gender | No human research participants used in this study. |
| Reporting on race, ethnicity, or other socially relevant groupings | No human research participants used in this study. |
| Population characteristics | No human research participants used in this study. |
| Recruitment | No human research participants used in this study. |
| Ethics oversight | No human research participants used in this study. |

Note that full information on the approval of the study protocol must also be provided in the manuscript.

# Field-specific reporting

Please select the one below that is the best fit for your research. If you are not sure, read the appropriate sections before making your selection.

☒ Life sciences ☐ Behavioural & social sciences ☐ Ecological, evolutionary & environmental sciences

For a reference copy of the document with all sections, see nature.com/documents/nr-reporting-summary-flat.pdf

# Life sciences study design

All studies must disclose on these points even when the disclosure is negative.

**Sample size**

In this study, we sought to assess protection from SARS-CoV-2 challenge after administrating RBD-62 through aerosol inhalation. We aimed to compare the protection provided relative to the untreated control. After SARS-CoV-2 challenge, the amount of viral RNA detectible in nasal swabs and bronchoalveolar lavage would be evalulated in both control groups and those that received RBD-62. We estimated the standard deviation in viral loads to be approximately 0.5 logs based on historical data from other NHP studies (1, 2). This number of animals (n=8/group) thus allowed us 80% power to detect differences in antibody titers that differ by 0.7 logs; this is sufficient for our study, in which we hypothesized a decrease of over 1.0 log.

1. Corbett, K.S., et al. Protection against SARS-CoV-2 Beta variant in mRNA-1273 vaccine-boosted nonhuman primates. Science 374, 1343-1353 (2021).
2. Gagne, M., et al. Mucosal Adenoviral-vectored Vaccine Boosting Durably Prevents XBB.1.16 Infection in Nonhuman Primates. bioRxiv, 2023.2011.2006.565765 (2023).

**Data exclusions**

No data was excluded from this study.

**Replication**

We used multiple experimental approaches to verify reproducibility of data including both sgRNA and TCID50 analysis for determination of virus replication, measurement of antigen-specific T cell responses using lymphocytes isolated from distinct compartments (blood & BAL), and measurement of humoral responses using antibodies from multiple compartments (blood, BAL, nasal wash / NW) and multiple related antigens (WT spike, RBD & N as well as Delta and BA.1 spikes). For determinations of drug potency, we calculated IC50 values for both ACE2 binding inhibition and inhibition of infection with authentic virus for a large panel of variants.

Additionally we measured virus RNA transcripts by performing two independent PCR assays (for different virus-specific targets). Although the amplified subgenomic transcript (E vs N) was unique to each PCR run (with differences in RNA copy number of ~1log in our prior experiments), and thus the virus RNA copy numbers in this experiment were also different between each PCR run (by ~1log), the kinetics and relative differences between the control and RBD-62 treated groups were nearly identical post-challenge between both assays.

Due to the use of these orthogonal approaches to validate our findings, most assays were run a single time. However, the in vitro determination of IC50 for ACE2 binding inhibition with soluble RBD-62 was performed three independent times. In each case, IC50 for RBD-62 against the ancestral variant was 1-3% of the IC50 value determined for wildtype RBD (ie, about 50x more potent).

Finally, we measured ACE2 inhibition twice using nasal washes from NHP post-challenge as an indication of a primary immune response. We were unable to clearly observe nasal wash inhibitory antibodies using this assay either time.

All attempts to repeat an experimental assay were successful and consistent with the reported data.

**Randomization**

All primates were stratified based on age and weight and randomized into either the control or treatment groups using an alternating pattern.

**Blinding**

Subgenomic RNA analysis, which was the primary endpoint, was conducted by scientists formally blinded to group status. For all other assays, scientists were not formally blinded as knowledge of which samples may have contained excess RBD-62 may have assisted in determination of which readouts expressed higher background levels, although, in practice, group status was largely unknown to investigators during

performance of experiments.

# Reporting for specific materials, systems and methods

We require information from authors about some types of materials, experimental systems and methods used in many studies. Here, indicate whether each material, system or method listed is relevant to your study. If you are not sure if a list item applies to your research, read the appropriate section before selecting a response.

## Materials & experimental systems

| n/a | Involved in the study |
| --- | --- |
| ☐ | ☒ Antibodies |
| ☐ | ☒ Eukaryotic cell lines |
| ☒ | ☐ Palaeontology and archaeology |
| ☐ | ☒ Animals and other organisms |
| ☒ | ☐ Clinical data |
| ☒ | ☐ Dual use research of concern |
| ☒ | ☐ Plants |

## Methods

| n/a | Involved in the study |
| --- | --- |
| ☒ | ☐ ChIP-seq |
| ☐ | ☒ Flow cytometry |
| ☒ | ☐ MRI-based neuroimaging |

## Antibodies

**Antibodies used**

We used the following antibodies for ICS with dilutions indicated in parentheses:
1. Live/dead fixable aqua dead cell stain, Invitrogen #L34957 – Lot #2204200 (1:800)
2. CD45RA PE-CY5, clone 5H9, BD Biosciences #552888 – Lot #8110737 (1:2500)
3. CD4 PE-CY5.5, clone S3.5, Invitrogen #MHCD0418 – Lot #2303833 (1:80)
4. ICOS PE-CY7, clone C398.4A, Biolegend #313520 – Lot #B213626 (1:640)
5. CD8 BV570, clone RPA-T8, Biolegend #301038 – Lot #B333843 (1:80)
6. CCR7 BV650, clone G043H7, Biolegend #353234 – Lot #B325079 (1:10)
7. CXCR3 BV711, clone 1C6/CXCR3, BD Biosciences #563156 – Lot #0309602 (1:20)
8. PD-1 BUV737, clone EH12.1, BD Horizon #612792 – Lot #0303349 (1:40)
9. TNF FITC, clone Mab11, BD Biosciences #554512 – Lot #0015360 (1:80)
10. IL-4 BB700, clone MP4-25D2, BD Biosciences custom order – Lot #1042139 (1:20)
11. CXCR5 PE, clone MU5UBEE, Thermo Fisher #12-9185-42 – Lot #2279157 (1:10)
12. CD69 ECD, clone TP1.55.3, Beckman Coulter #6607110 – Lot #7620090 (1:40)
13. IL-21 Ax647, clone 3A3-N2.1, BD Biosciences #560493 – Lot #1005849 (1:10)
14. IFN-g Ax700, clone B27, Biolegend #506516 – Lot #B320892 (1:640)
15. CD3 APC-CY7, clone SP34.2, BD Biosciences #557757 – Lot #0223215 (1:640)
16. IL-13 BV421, clone JES10-5A2, BD Biosciences #563580 – Lot #0286560 (1:20)
17. IL-17A BV605, clone BL168, Biolegend #512326 – Lot #B319897 (1:40)
18. CD154 BV785, clone 24-31, Biolegend #310842 – Lot #B329207 (1:20)
19. IL-2 BV750, clone MQ1-17H12, BD Biosciences #566361 – Lot #7108833 (1:40)

We used the following antibodies for B cell analysis:
1. Live/dead fixable aqua dead cell stain, Invitrogen #L34957 – Lot #2098529 (1:800)
2. IgD FITC, goat pAb, Southern Biotech #2030-02 – Lot #A2118-WF09C (1:40)
3. IgM PerCP-Cy5.5, clone G20-127, BD Biosciences #561285 – Lot #0307134 (1:40)
4. IgA Dy405, goat pAb, Jackson ImmunoResearch #109-475-011 – Lot #155196 (1:40)
5. CD20 BV570, clone 2H7, Biolegend #302332 – Lot #B301458 (1:40)
6. CD27 BV650, clone O323, Biolegend #302828 – Lot #B273921 (1:20)
7. CD14 BV785, clone M5E2, Biolegend #301840 – Lot #B327948 (1:80)
8. CD8 BUV395, clone RPA-T8, BD Biosciences #563795 – Lot #9346411 (1:80)
9. CD16 BUV496, clone 3G8, BD Biosciences #564653 – Lot #0288806 (1:40)
10. CD4 BUV737, clone SK3, BD Biosciences #564305 – Lot #0282762 (1:40)
11. CD19 APC, clone J3-119, Beckman Coulter #IM2470U – Lot #200092 (1:20)
12. IgG Ax700, clone G18-145, BD Biosciences #561296 – Lot #0135021 (1:20)
13. CD3 APC-Cy7, clone SP34.2, BD Biosciences #557757 – Lot #0223215 (1:40)
14. CD38 PE, clone OKT10, Caprico Biotech #100826 – Lot #8AE4 (1:640)
15. CD21 PE-Cy5, clone B-ly4, BD Biosciences #551064 – Lot #0072939 (1:20)
16. CXCR5 PE-Cy7, clone MU5UBEE, Thermo Fisher #25-9185-42 – Lot #2312036 (1:40)

We used the following antibody for in vitro infection assay:
1. SARS-CoV-2 spike protein human IgG1 mAb Alexa 647, clone CR3022, Cell Signaling #37475 (1:5000)

**Validation**

Validation for ICS assay is described previously {Donaldson, M.M., Kao SF, Foulds KE. OMIP-052: An 18-Color Panel for Measuring Th1, Th2, Th17, and Tfh Responses in Rhesus Macaques. Cytometry A 95, 261-263 (2019)}. All antibodies are advertised to work against human except CXCR5 (clone MU5UBEE) which is raised against Rhesus Macaques.

All antibodies for B- and T-cell assays are titrated, per lot, on PBMC from rhesus macaques for optimal staining condition and then in the context of the full panel for optimization.

Antibody used for measurement of virus for in vitro inhibition was previously described in the following publications as listed on the manufacturer's website:
1. Suzuki, Y. et al. Design and lyophilization of lipid nanoparticles for mRNA vaccine and its robust immune response in mice and nonhuman primates. Mol. Ther. Nucleic Acids 30, 226-240 (2022).
2. Barber, K.W., Shrock, E. & Elledge, S.J. CasPlay provides a gRNA-barcoded CRISPR-based display platform for antibody repertoire profiling. Cell Rep. Methods 2, 100318 (2022).

## Eukaryotic cell lines

Policy information about cell lines and Sex and Gender in Research

| | |
|---|---|
| Cell line source(s) | For TCID50 analysis and in vitro infection assay, VeroE6-TMPRSS2 cells were obtained from the Vaccine Research Center, NIH. Vero cells are assumed to have been isolated from a female African green monkey and are the standard cell line used for SARS-CoV-2 virus culture and neutralization assays. |
| Authentication | Cell lines were authenticated by characterization of TMPRSS2 via use of an anti-TMPRSS2 flow antibody.. |
| Mycoplasma contamination | All cell lines tested negative for mycoplasma contamination. |
| Commonly misidentified lines (See ICLAC register) | No commonly misidentified cell lines used in this study. |

## Animals and other research organisms

Policy information about studies involving animals; ARRIVE guidelines recommended for reporting animal research, and Sex and Gender in Research

| | |
|---|---|
| Laboratory animals | For this study, we enrolled four- to seven-year-old male Indian-origin rhesus macaques (Macaca mulatta). |
| Wild animals | This study did not involve wild animals. |
| Reporting on sex | All primates were male. We only analyzed male primates as disease severity is more pronounced in male populations (for both humans and animal models) and thus would provide a higher threshold for determination of any protective effect arising from RBD-62 treatment. |
| Field-collected samples | No samples were collected from the field. |
| Ethics oversight | Animals were housed at Bioqual, Inc. All experiments were conducted according to National Institutes of Health (NIH) standards on the humane care and use of laboratory animals, and all procedures approved by and conducted in accordance with regulations of the Animal Care and Use Committees of the NIH Vaccine Research Center (VRC) and BIOQUAL, Inc. (Rockville, Maryland). Animals were housed and cared for in accordance with local, state, federal and institute policies in facilities accredited by the American Association for Accreditation of Laboratory Animal Care (AAALAC), under standards established in the Animal Welfare Act and the Guide for the Care and Use of Laboratory Animals. |

Note that full information on the approval of the study protocol must also be provided in the manuscript.

## Flow Cytometry

### Plots

Confirm that:

☒ The axis labels state the marker and fluorochrome used (e.g. CD4-FITC).

☒ The axis scales are clearly visible. Include numbers along axes only for bottom left plot of group (a 'group' is an analysis of identical markers).

☒ All plots are contour plots with outliers or pseudocolor plots.

☒ A numerical value for number of cells or percentage (with statistics) is provided.

### Methodology

| | |
|---|---|
| Sample preparation | All tissue are rhesus macaque in origin. BAL cells were obtained by PBS lavage under anesthesia. Cells were quantified using Nexcelom Cellaca cell counter, cryopreserved in 90% FBS, 10% DMSO, using a controlled rate freezer (Thermo Fisher Scientific). |
| Instrument | BD FACSymphony X-50 (LSR Fortessa), Model Number N/A – Special Order Research Product. REF 660964 |
| Software | We used BD FACS Diva for acquisition. We performed analysis and data export in BD FlowJo 10 (version 10.7.2 for B cell binding or version 10.8.2 for ICS). Excel was used for data sorting and background subtraction while Graphpad Prism 9 was used for graphical figures. |

| | |
|---|---|
| Cell population abundance | No sorting was performed, but for PBMC T-cell assays, 35% viability threshold was applied for exclusion criteria. For BAL T cell assay, samples with less than 5,000 live CD3 were excluded. No exclusion criteria was specified for B cell assay. |
| Gating strategy | Boundaries between positive and negative staining cell populations were defined based on stained negative control samples. Specifically, positive populations were defined by stimulated (using spike or nucleoprotein peptides) vs. unstimulated (DMSO) conditions (for T-cell assay) or pre-exposure vs. post-challenge conditions (for B-cell assay). Details on gating strategy are listed below.<br>For B cell assay: Cells were gated as singlets and live cells on forward and side scatter and a live/dead aqua blue stain. Cells were further gated based on lack of expression of CD3, CD4, CD14 and CD16. B cells were then defined based on expression of CD20 and CD19 whereas memory B cells were gated based on lack of IgD or IgM expression. Finally variant S-2P probe pairs were used to define binding specificity.<br>For ICS: Cells were gated as singlets and live cells on forward and side scatter and a live/dead aqua blue stain. CD3+ events were gated as CD4+ or CD8+ T cells. Total memory CD8+ T cells were selected based on expression of CCR7 and CD45RA. Finally, SARS-CoV-2 S-specific memory CD8+ T cells were gated according to co-expression of CD69 and IL-2, TNF or IFNγ. The CD4+ events were defined as total memory or central memory according to expression of CCR7 and CD45RA. CD4+ cells with a TH1 phenotype were defined as memory cells that coexpressed CD69 and IL-2, TNF or IFNγ. CD4+ cells with a TH2 phenotype were defined as memory cells that co-expressed CD69 and IL-4 or IL-13. TFH cells were defined as central memory CD4+ T cells that expressed CXCR5, ICOS and PD-1. TFH cells were further characterized as IL-21+, CD69+ or CD40L+, CD69+. |

☒ Tick this box to confirm that a figure exemplifying the gating strategy is provided in the Supplementary Information.
